# Cell-penetrating peptide-grafted AAV2 capsids for improved retinal delivery via intravitreal injection

**DOI:** 10.1016/j.omtm.2025.101426

**Published:** 2025-02-03

**Authors:** Jiang-Hui Wang, Mengtian Cui, Hao Liu, Peiyi Guo, Jackson McGowan, Shun-Yun Cheng, Dominic J. Gessler, Jun Xie, Claudio Punzo, Phillip W.L. Tai, Guangping Gao

**Affiliations:** 1 Horae Gene Therapy Center, University of Massachusetts Medical School, Worcester, MA 01605, USA; 2 Department of Microbiology and Physiological Systems, University of Massachusetts Chan Medical School, Worcester, MA 01605, USA; 3 Centre for Eye Research Australia, Royal Victorian Eye and Ear Hospital, East Melbourne, VIC 3002, Australia; 4 Ophthalmology, Department of Surgery, University of Melbourne, East Melbourne, VIC 3002, Australia; 5 Department of Ophthalmology and Visual Sciences, University of Massachusetts Chan Medical School, Worcester, MA 01605, USA; 6 Department of Neurological Surgery, University of Massachusetts Chan Medical School, Worcester, MA 01605, USA; 7 Department of Neurosurgery, University of Minnesota, Minneapolis, MN 55455, USA; 8 Li Weibo Institute for Rare Diseases Research, University of Massachusetts Medical School, Worcester, MA 01605, USA

**Keywords:** AAV2, cell-penetrating peptide, capsid screening, photoreceptor, intravitreal injection

## Abstract

Recombinant adeno-associated virus (rAAV) is a leading vector for retinal gene therapy due to its favorable safety profile demonstrated by the FDA-approved Luxturna for Leber congenital amaurosis. However, challenges with low transduction efficiency and immunogenicity, coupled with the invasiveness of subretinal injections, have driven efforts to engineer AAV capsids for minimally invasive intravitreal delivery. Intravitreal injections face the barrier of the inner limiting membrane (ILM), particularly with AAV2-based vectors. In this study, we displayed cell-penetrating peptides (CPPs) on AAV2 capsids to enhance retinal cell transduction via intravitreal injection. Through *in vivo* capsid screening, we identified AAV2.CPP1, which showed significantly improved pan-retinal expression and photoreceptor transduction in mice as well as a reduced immune response compared to the AAV2.7m8 vector. We also revealed that the CPP1 insertion reduced heparan sulfate binding, improving ILM penetration. These findings highlight AAV2.CPP1 as a promising candidate for retinal gene therapy via intravitreal injection, offering enhanced efficiency and a minimized immune response.

## Graphical abstract

**Figure:**
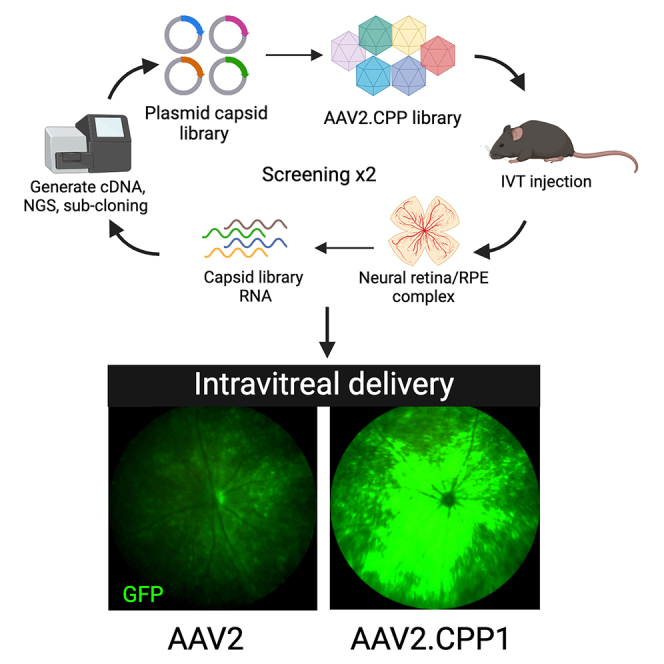

## Teaser

Engineering AAV2 capsids with a cell-penetrating peptide (CPP1) enhances intravitreal retinal gene delivery by improving inner limiting membrane penetration, photoreceptor transduction, and pan-retinal expression while reducing immune responses. This study identifies AAV2.CPP1 as a promising candidate for minimally invasive retinal gene therapy with improved efficiency and safety.

## Introduction

Recombinant adeno-associated virus (rAAV) has emerged as the preferred delivery vector for both experimental and clinical gene therapies, particularly for treating inherited retinal diseases (IRDs), due to its favorable safety profile.^1^^,^^2^^,^^3^ Following the landmark approval by the US Food and Drug Administration (FDA) of Luxturna, the first rAAV-based gene therapy product for Leber congenital amaurosis type 2 (LCA2), many rAAV-based clinical trials for other IRDs are currently underway.^3^^,^^4^ IRDs primarily cause degeneration of two outer retinal cell types, including photoreceptors and retinal pigment epithelium (RPE) cells, due to various genetic mutations. Both cells play crucial roles in phototransduction and retina metabolism.^5^ Current rAAV-based gene therapy mainly targets these two cell types through subretinal injection.

However, concerns have arisen regarding the low transduction efficacy and vector-induced immunogenicity.^6^ In addition, subretinal injection, while effective, is invasive and transduces only a limited number of photoreceptors and RPEs, reducing overall therapeutic efficacy. This has led to efforts to engineer novel capsids capable of transducing photoreceptors and RPEs via intravitreal injection, a minimally invasive route that provides access to the entire retina. AAV2 has been widely used in ocular gene therapy due to its strong tropism for retinal cells, high transduction efficiency, and established safety profile in clinical studies. Despite this, the major limitation for intravitreal injection of rAAV2-based vectors is its inability to transduce photoreceptors and RPEs, potentially due to strong binding of heparan sulfate (HS) proteoglycans (HSPGs) enriched in the inner limiting membrane (ILM), a major structure barrier for rAAV2-based ocular gene therapy through the intravitreal route.^7^ One promising development is the engineered AAV2 capsid 7m8, which has demonstrated the capability to transduce photoreceptors via the intravitreal route in mice.^8^ 7m8 was discovered through directed evolution-based screening of a seven-amino-acid library inserted at the 3-fold spike region of AAV2, specifically N587 of VP1. However, its robust photoreceptor transduction observed in mice was not replicated in non-human primates (NHPs), where transduction was limited to the macula and peripheral retina. This was likely due to the absence of or significantly thinner ILM in these regions compared to the central retina.^9^

Cell-penetrating peptides (CPPs) are short amino acid sequences, often rich in positively charged residues like arginine, which enable them to interact with and penetrate negatively charged cell membranes.^10^ Originally inspired by viral proteins like TAT, CPPs have been synthetically engineered to enhance the delivery of therapeutic molecules such as drugs and genes. Cationic CPPs like polyarginine enter cells through mechanisms such as micropinocytosis or direct membrane interaction, while amphiphilic CPPs leverage both hydrophilic and hydrophobic regions to exploit the amphipathic nature of cell membranes for cargo delivery.^11^ These peptides can be optimized for enhanced uptake, stability, and reduced toxicity. In ocular drug delivery, CPPs present a promising non-invasive alternative as eye drops to repeated intravitreal injections for conditions such as age-related macular degeneration (AMD). A study has shown that CPP-conjugated anti-vascular endothelial growth factor drugs delivered topically can be as effective as intravitreal injections in AMD animal models.^12^ A similar approach in designing AAV9 variants with CPPs resulted in two variants, AAV.CPP.16 and AAV.CPP.21, demonstrating improved transduction of CNS cells in both mice and macaques, showing promise for delivering therapeutic payloads such as in glioblastoma models.^13^ However, whether modifying AAV2, a commonly used vector in retinal gene therapy, with CPPs can enhance retinal cell transduction via intravitreal injection remains unclear.

Displaying random 7-mer peptides on the capsid is a standard technique in AAV capsid engineering via directed evolution. This method has led to the development of several tissue-specific, high-performing capsids, such as AAV.PHP.B and AAV.PHP.eB for CNS transduction^14^^,^^15^ and AAV2.7m8 for retinal applications.^8^ To date, no study has utilized peptides of varying lengths for capsid bio-panning *in vivo*, likely due to the inherent limitations in variant diversity and the challenges related to the efficient synthesis and cloning of DNA libraries and the tolerability of longer peptide insertions within the capsid. In this study, we utilized a combined rational design and semi-directed evolution approach, efficiently constructing a library of AAV2 capsid variants featuring a wide range (3- to 42-mer) of known CPPs with varying lengths inserted at HSPG-binding regions. The library underwent two rounds of screening via intravitreal injection in mice, leading to the identification of AAV2.CPP1 (a 5-mer insertion), a variant with enhanced pan-retinal transduction and reduced immune response compared to AAV2.7m8. Notably, AAV2.CPP1 demonstrated superior photoreceptor transduction. We revealed that the CPP1 insertion reduced HS binding, likely facilitating greater vector penetration through the ILM and more effective retinal cell transduction. These results suggest that AAV2.CPP1 holds promise for AAV-based retinal gene therapies via intravitreal injection.

## Results

### Development and *in vivo* evaluation of AAV2 capsid libraries featuring CPPs and photoreceptor-targeted vectors

We hypothesized that grafting CPPs onto AAV2 capsids could similarly enhance retinal cell transduction following intravitreal injection. To test this hypothesis, we curated a CPP library by selecting peptides with unique amino acid sequences from the CPPsite2.0 database,^16^ which contains approximately 1,700 unique CPPs. The corresponding DNA sequences were synthesized, generating a library of 1,090 CPPs, ranging from 3-mer to 42-mer, confirmed by next-generation sequencing (NGS) and ready for cloning (Table S1).

**Figure 1:**
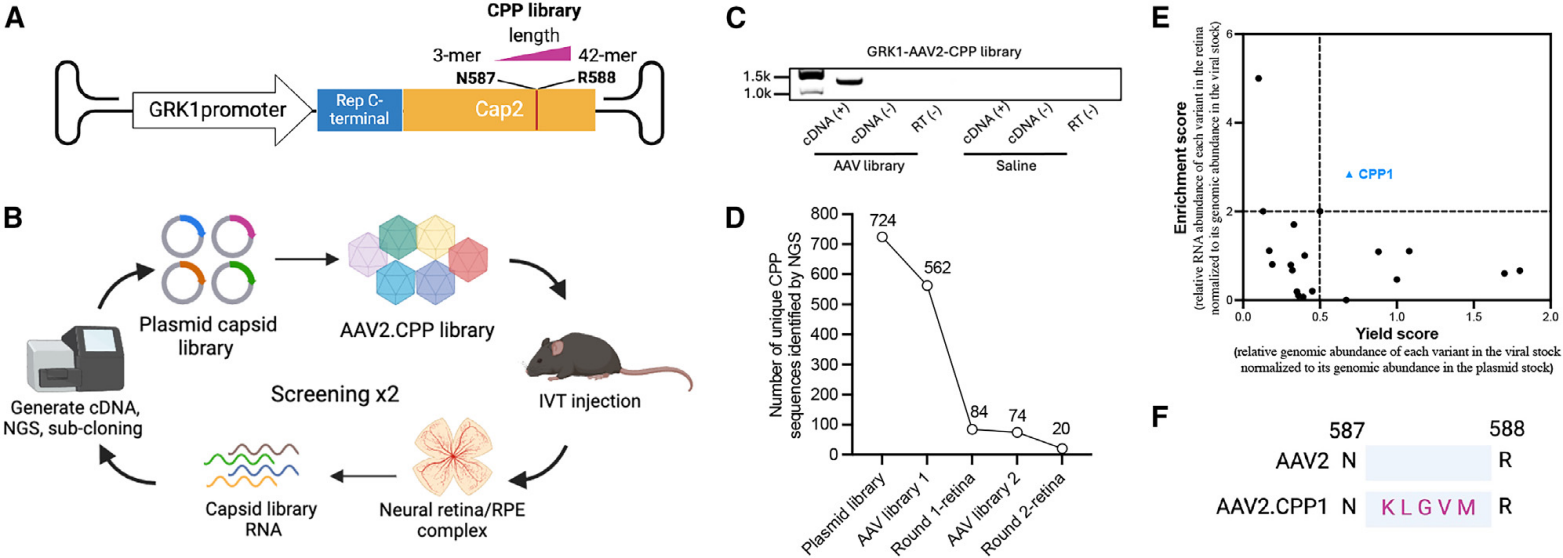
Identification of the leading AAV2 capsid variant enriched in the retina of mice (A) Schematic representation of the backbone plasmid construct containing the cell-penetrating peptide (CPP) library insert. (B) Workflow of the screening process in mice, involving two rounds of selection. (C) Representative RT-PCR results of recovered RNA of capsid variants from pooled retina/RPE tissues 28 days after injection of the GRK1-driven AAV library. (D) The reduction and enrichment of CPP variants during each round of selection. (E) Identification of AAV2.CPP1 as the leading capsid variant after the second round of selection based on enrichment and yield scores. The enrichment score refers to the relative RNA abundance of each variant in the retina, normalized to its genomic abundance in the viral stock. The yield score represents the relative genomic abundance of each variant in the viral stock, normalized to its genomic abundance in the plasmid stock. (F) Sequence of the peptide insert on the AAV2.CPP1 capsid. CPP, cell-penetrating peptides; RT, reverse transcriptase.

Following a method previously described by Nonnenmacher et al.,^17^ we constructed a diverse capsid library by grafting the CPP sequences between residues 587 and 588 in the hypervariable surface loop VIII of AAV2 capsid genes by Gibson assembly, which was combined with AAV2 ITRs and a photoreceptor-specific G-protein-coupled receptor kinase 1 (GRK1) promoter (Figure 1A). This site permits genetic modifications without disrupting capsid assembly or genome packaging.^18^ The resulting plasmid library retained 66.4% (724/1,090) of the CPPs from the initial library. Rep proteins were provided via a separate plasmid during virus production. Viral libraries were generated in HEK293T cells under low-DNA-input conditions to minimize capsid mosaicism and cross-packaging.^19^ The viral libraries were intravitreally injected into both eyes of adult C57BL/6 mice (*n* = 5). After 28 days, the retina and RPE complex were harvested and processed, and capsid library sequences were recovered by RT-PCR (Figure 1B). Amplicon pools were recloned into GRK1 AAV vectors for a second round of selection. A limited number of capsid amplicons were detected in retinal samples from mice injected with the AAV libraries, as evidenced by the relatively weak intensity of the expected band on the gel (Figure 1C). In contrast, saline-injected controls showed no detectable amplicons.

NGS analysis was conducted after each round of *in vivo* selection to assess variant diversity and enrichment in the retina. The first round reduced the number of unique sequences from 724 (plasmid library) to 84 (retinal tissue), eliminating 88% of the variants. In the second round, 76% of the remaining variants were removed, reducing the pool to 20 unique sequences in the retinal tissue (Figures 1D and S1; Table 1). Interestingly, many CPP sequences were absent following AAV packaging, suggesting that certain CPPs with variable length and sequences may interfere with VP3 protein structure and function, rendering them non-packageable. We analyzed enrichment and AAV packaging efficiency after the second round of screening, using an enrichment score threshold of >2.0 and a yield score >0.5. The results identified a distinct CPP sequence, KLGVM, which met the selection criteria and was designated AAV2.CPP1 (Figure 1E). To validate these findings, we cloned the original CPP library into an AAV genome driven by the CB6 promoter (CMV enhancer/CB promoter) and performed two rounds of *in vivo* selection in adult C57BL/6 mice via intravitreal injection. Remarkably, AAV2.CPP1 consistently emerged as the top candidate, meeting the selection criteria (Figures 1F and S2).

**Table 1:** The CPP sequences identified in the mouse retina after the second-round screen

Amino acid sequence of the leading CPP	Enrichment score	Yield score
KLGVM (CPP1)	2.84	0.69
PNTRVRPDVSF	2.00	0.13
VPTLK	2.00	0.50
VRLPPP	1.70	0.33
SYIQRTPSTTLP	1.11	0.17
FKQQQQQQQQQQ	1.10	1.08
VKLPPP	1.09	0.88
KLPVM	1.00	0.40
NSGTMQSASRAT	0.80	0.19
TFPQTAIGVGAP	0.79	0.31
VSALK	0.67	0.32
TSHTDAPPARSP	0.66	1.80
PMLKE	0.60	1.70
DRDRDRDRDR	0.46	1.00
PSSSSSSRIGDP	0.20	0.45
SPMQKTMNLPPM	0.19	0.35
NHQQQNPHQPPM	0.10	0.36
MLKTTELLKTTELLKTTE	0.07	0.39
VPTLQ	0.00	0.67

### Enhanced photoreceptor transduction by single-stranded AAV2.CPP1 in mice via intravitreal injection

We packaged several batches of rAAVs using AAV2, AAV2.7m8, and AAV2.CPP1 capsids. These carried either single-stranded (ss) genomes encoding cytoplasmic green fluorescent protein (*GFP*) driven by the CB6 promoter (ssAAV2.CB6.*GFP*, ssAAV2.7m8.CB6.*GFP*, and ssAAV2.CPP1.CB6.*GFP*) or histone H2B-tagged GFP (nuclear-localized *H2BGFP*) under the control of the GRK1 promoter (ssAAV2.GRK1.*H2BGFP*, ssAAV2.7m8.GRK1.*H2BGFP*, and ssAAV2.CPP1.GRK1.*H2BGFP*). In addition, we packaged self-complementary (sc) AAVs encoding cytoplasmic *GFP* (scAAV2.CB6.*GFP*, scAAV2.7m8.CB6.*GFP*, and scAAV2.CPP1.CB6.*GFP*). Vectors packaged with the CPP1 capsid showed an approximately 4-fold increase in viral titers compared to AAV2, regardless of whether the genome was single stranded or self-complementary (Figure S3A). This suggests that the insertion of the CPP1 peptide does not interfere with AAV production. To evaluate the integrity of rAAV particles purified by iodixanol gradient purification, we utilized high-resolution transmission electron microscopy (TEM) to examine the morphology of the virions (Figure S3B). A semi-quantitative assessment was performed by counting empty/partially full and fully packaged virions across six representative fields.^20^ The analysis revealed that the full-to-empty ratio for scAAV2.CPP1.CB6.GFP (∼60%) was comparable to that of scAAV2.CB6.GFP (∼70%) (Figure S3C).

**Figure 2:**
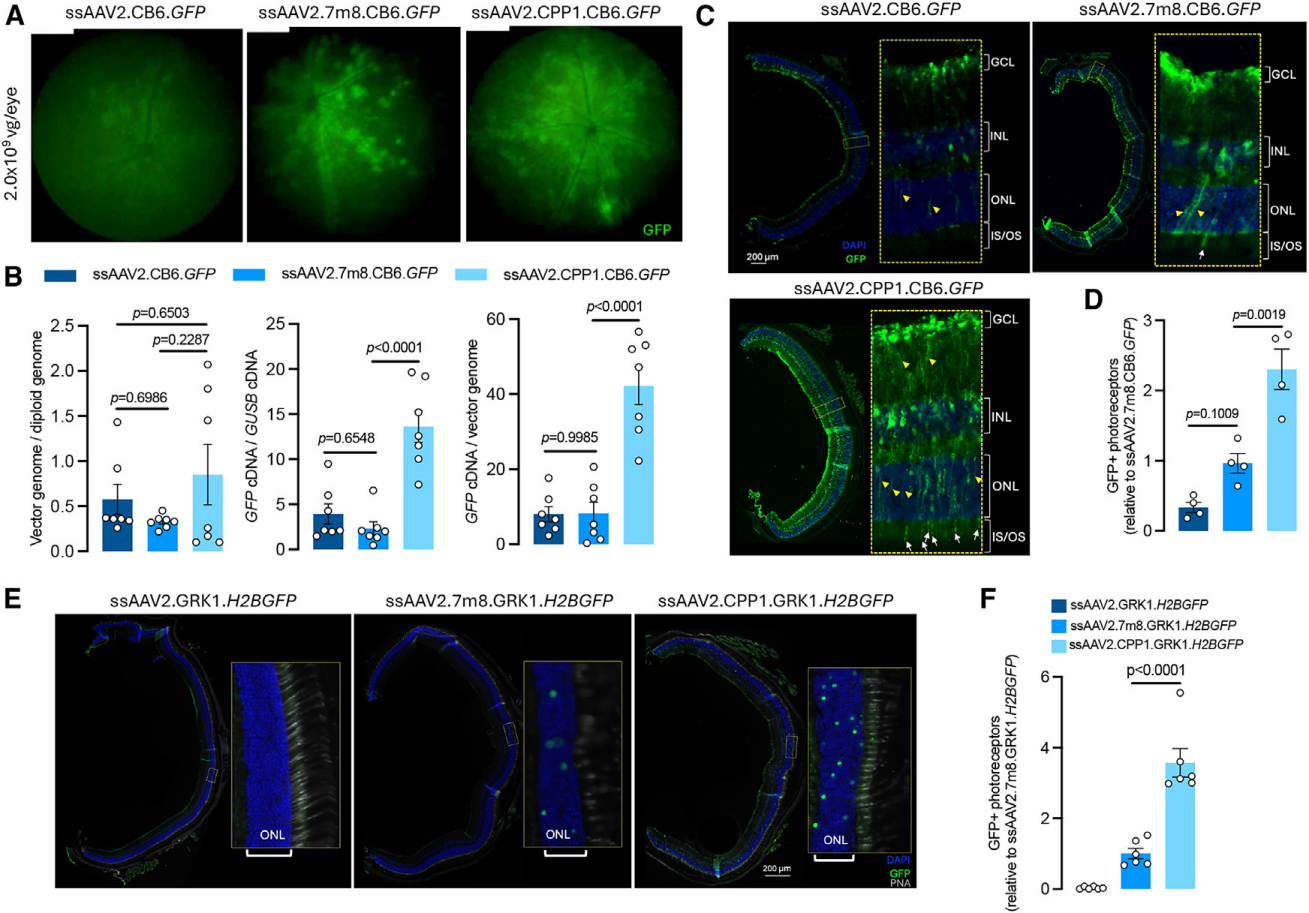
Retinal transduction profile of the single-stranded AAV2.CPP1 vector in adult mice (A) Representative fluorescence fundus images of adult C57BL/6 mice 4 weeks after intravitreal injection with a high dose (2.0 × 10^9^ vg/eye) of single-stranded AAVs. (B) Quantification of genomic DNA and mRNA expression levels of GFP in mouse retinas 4 weeks post-injection of high-dose ssAAVs. (C) Immunostaining of retinal cross sections 4 weeks after high-dose intravitreal injection of ssAAV2.CB6.GFP, ss7m8.CB6.GFP, and ssAAV2.CPP1.CB6.GFP. The white arrow shows the GFP expression in the inner segments (ISs) or outer segments (OSs) of photoreceptors successfully transduced. The yellow arrowheads indicate the transduced Müller cells. (D) Quantitative analysis of the number of transduced photoreceptors in retinas treated with different viral vectors. (E) Immunostained retinal cross sections showing nuclear GFP expression in photoreceptors 4 weeks after high-dose intravitreal injection of ssAAV2.GRK1.*H2BGFP*, ss7m8.GRK1.*H2BGFP*, and ssAAV2.CPP1.GRK1.*H2BGFP*. Peanut agglutinin (PNA) is a marker of photoreceptor OSs. ONL, outer nuclear layer. (F) Quantitative analysis of transduced photoreceptors in retinas treated with the indicated vectors.

To evaluate the retinal transduction efficiency, ssAAV2.CB6.*GFP*, ssAAV2.7m8.CB6.*GFP*, and ssAAV2.CPP1.CB6.*GFP* were intravitreally injected (2.0 × 10^9^ viral genomes [vg]/eye) into 2-month-old male C57BL/6 mice (*n* = 5–11). Four weeks post-injection, *in vivo* fluorescence fundus imaging was performed to assess GFP expression. ssAAV2.CPP1.CB6.*GFP* exhibited robust, homogeneous GFP expression throughout the retina, in contrast to the more localized, blood-vessel-centric GFP expression observed with ssAAV2.7m8.CB6.*GFP* and the weaker GFP signal seen with ssAAV2.CB6.*GFP* (Figure 2A).

Following imaging, retinal tissues were harvested for DNA biodistribution, RNA expression analysis, and immunohistology. ssAAV2.CPP1.CB6.*GFP* displayed DNA biodistribution similar to that of ssAAV2.CB6.*GFP* and ssAAV2.7m8.CB6.*GFP*. However, it resulted in significantly higher GFP expression at RNA levels, increasing by 3.5- and 5.8-fold, respectively. This divergence between DNA and RNA levels indicates that the improved transgene expression by ssAAV2.CPP1.CB6.*GFP* was primarily driven by enhanced transcription rather than biodistribution. The elevated RNA/DNA ratios further support this hypothesis (Figure 2B). At a lower dose of 2.0 × 10^8^ vg/eye, moderate increases in GFP expression were observed in mice receiving ssAAV2.CPP1.CB6.*GFP* via fluorescence fundus imaging; however, no significant differences at RNA levels were detected between the vectors (Figure S4).

Immunohistological analysis of GFP expression in retinal cross sections confirmed that ssAAV2.CPP1.CB6.*GFP* achieved uniform transduction across most retinal cell types and exhibited stronger GFP expression compared to ssAAV2.CB6.*GFP* and ssAAV2.7m8.CB6.*GFP*, which showed weaker and more patchy expression patterns (Figure 2C). These results align with the fundus imaging findings, confirming that ssAAV2.CPP1.CB6.*GFP* delivers pan-retinal transduction. Importantly, ssAAV2.CPP1.CB6.*GFP* transduced approximately 2.5 times more photoreceptors, as indicated by GFP expression in their inner or outer segments, compared to ssAAV2.7m8.CB6.*GFP* (Figure 2D), a well-known vector for effective outer retinal cell transduction via intravitreal injection, while ssAAV2 rarely transduced photoreceptors. We also observed a significantly higher number of Müller cells transduced by ssAAV2.CPP1.CB6.*GFP* compared to other vectors, as indicated by its extended radial processes spanning the retina from the ganglion cell layer to the outer nuclear layer (Figure 2C).

Given the challenges in assessing cytoplasmic GFP expression in photoreceptors by quantifying the number of outer segments expressing GFP, we developed a new AAV genome encoding nuclear-localized H2BGFP, driven by the GRK1 promoter, and packaged it into AAV2, AAV2.7m8, and AAV2.CPP1 capsids (ssAAV2.GRK1.*H2BGFP*, ssAAV2.7m8.GRK1.*H2BGFP*, and ssAAV2.CPP1.GRK1.*H2BGFP*). This approach allows for clearer and more straightforward assessment of H2BGFP expression specifically in photoreceptor nuclei (Figure 2E). Upon intravitreal injection, H2BGFP was homogeneously expressed in the photoreceptor nuclei of eyes treated with ssAAV2.7m8.GRK1.*H2BGFP* and ssAAV2.CPP1.GRK1.*H2BGFP*, but not in those treated with ssAAV2.GRK1.*H2BGFP*. Quantitative analysis showed that ssAAV2.CPP1.GRK1.*H2BGFP* increased photoreceptor transduction nearly 4-fold compared to ssAAV2.7m8.GRK1.*H2BGFP*.

### Enhanced photoreceptor transduction by scAAV2.CPP1 in mice via intravitreal injection

**Figure 3:**
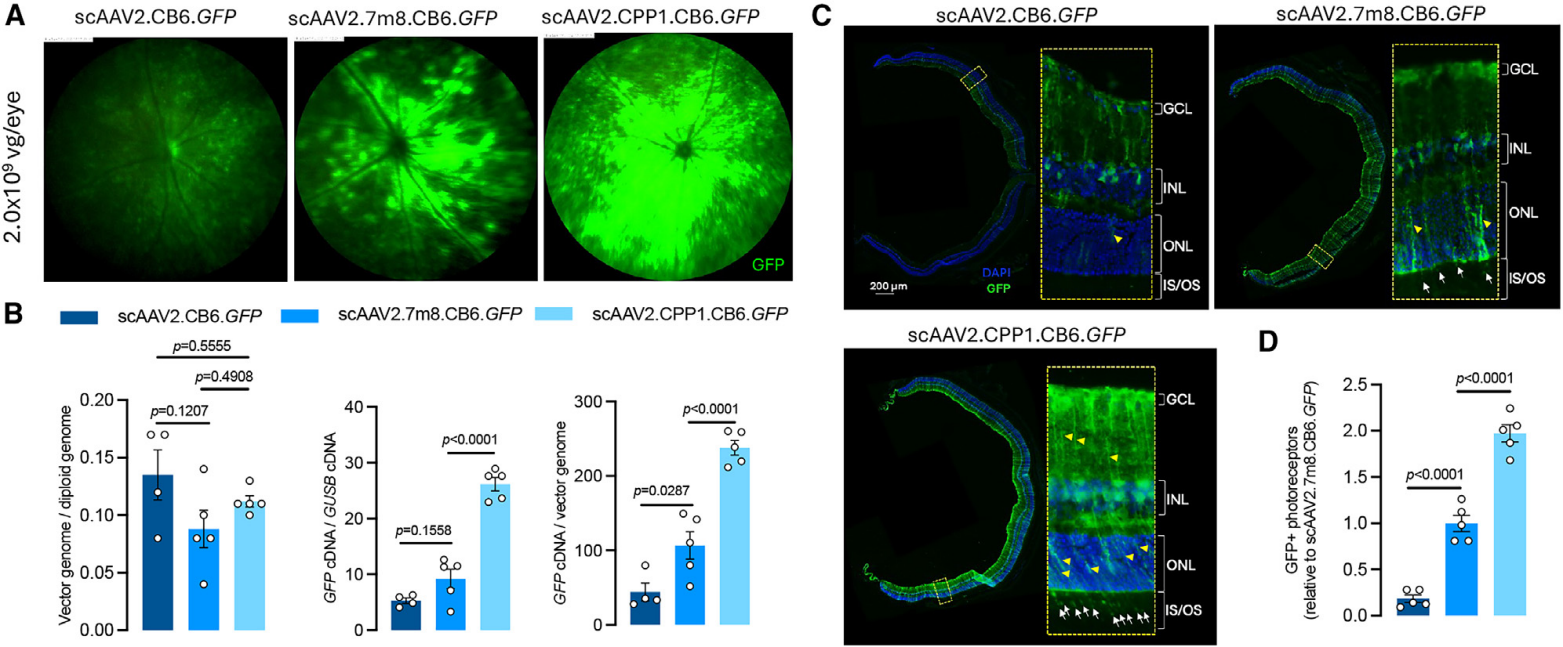
Retinal transduction profile of self-complementary AAV2.CPP1 vector in adult mice (A) Fundus images showing retinal transduction 4 weeks after intravitreal (IVT) injection of a high dose (2.0 × 10^9^ vg/eye) of self-complementary (sc) AAVs in adult mice. (B) Quantification of genomic DNA and mRNA expression levels of GFP in the mouse retina 4 weeks post-injection of high-dose scAAVs. (C) Immunostained retinal cross sections from mice injected with high-dose scAAV2.CB6.*GFP*, sc7m8.CB6.*GFP*, and scAAV2.CPP1.CB6.*GFP*. (D) Quantitative analysis of transduced photoreceptors in retinas treated with different viral vectors.

To evaluate the transduction capability of the scAAV2.CPP1, we administered intravitreally 2.0 × 10^9^ vg/eye of scAAV2.CB6.GFP, scAAV2.7m8.CB6.GFP, and scAAV2.CPP1.CB6.*GFP* in mice. Fundus imaging revealed a similar expression pattern in scAAV2.CPP1-treated mice, but with significantly higher GFP expression compared to the single-stranded vectors (Figure 3A). Molecular analysis of transgene expression corroborated the findings from the single-stranded vectors. While all three vectors exhibited similar DNA biodistributions in the retina (Figure 3B), scAAV2.CPP1.CB6.GFP showed a 5- and 2.9-fold increase in GFP expression at the RNA level compared to scAAV2.CB6.*GFP* and scAAV2.7m8.CB6.*GFP*, respectively. The increase in RNA/DNA ratios of scAAV2.CPP1.CB6.*GFP* was consistent with the results observed for the single-stranded vectors.

Immunohistological analysis showed that GFP expression was more uniform and pronounced with scAAV2.CPP1.CB6.*GFP* than with the other vectors (Figure 3C). As expected, photoreceptor transduction was notable in eyes receiving scAAV2.7m8.CB6.*GFP*, but scAAV2.CPP1.CB6.*GFP* led to even more extensive transduction. Quantitative analysis confirmed that scAAV2.CPP1.CB6.*GFP* transduced twice as many photoreceptors as scAAV2.7m8.CB6.*GFP* (Figure 3D). Similar to Figure 2C, we observed enhanced transduction of Müller cells by scAAV2.CPP1.CB6.*GFP* (Figure 3C). We also assessed GFP expression at a lower dose (2.0 × 10^8^ vg/eye). Although fundus imaging showed an increase in GFP expression with both scAAV2.7m8.CB6.*GFP* and scAAV2.CPP1.CB6.*GFP* compared to that with scAAV2.CB6.*GFP*, no significant differences at the RNA level were observed among the vectors (Figure S5).

### Decreased immune responses by AAV2.CPP1 vector

**Figure 4:**
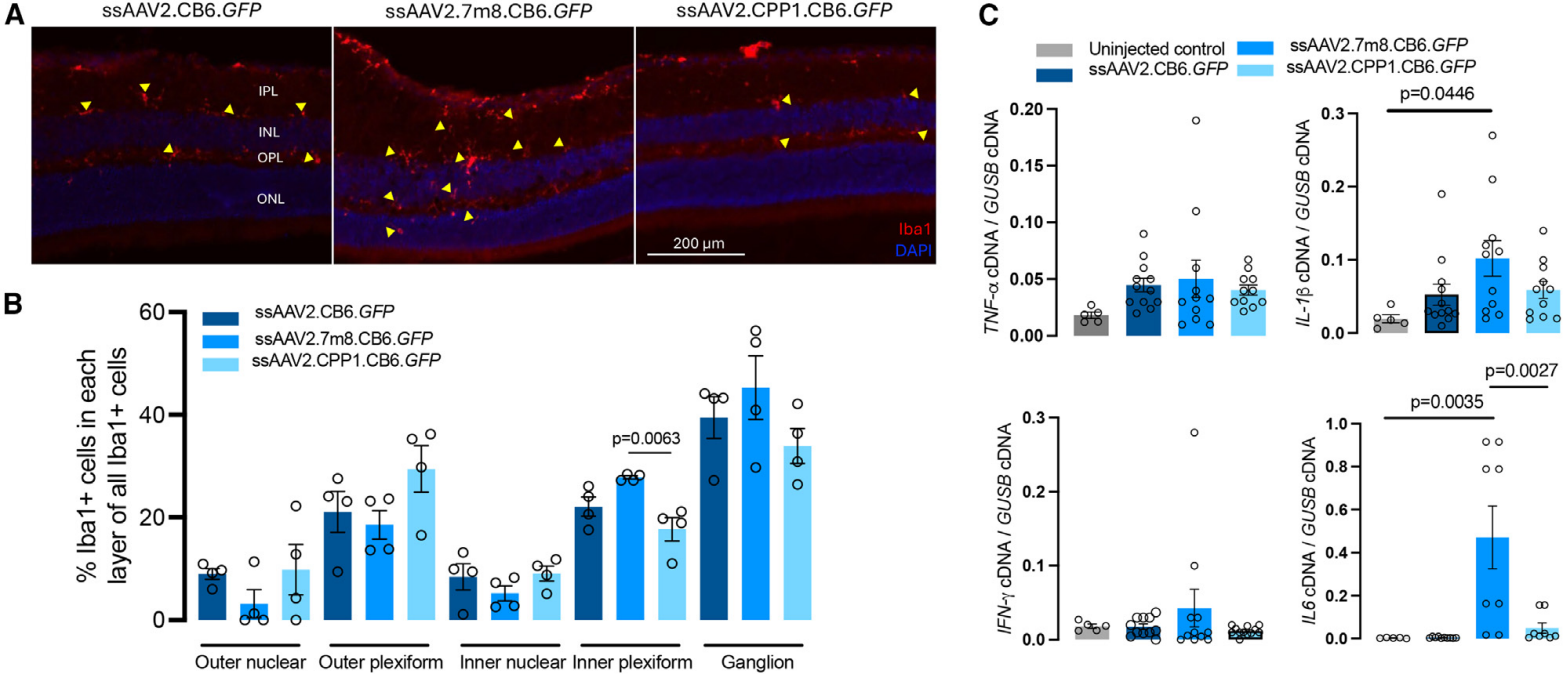
Minimal immune response induced by the AAV2.CPP1 vector compared to AAV2.7m8 (A) Representative immunostaining of retinal microglia using the IBA1 marker in eyes treated with ssAAV2.CB6.*GFP*, ss7m8.CB6.*GFP*, and ssAAV2.CPP1.CB6.*GFP*. (B) Quantification of IBA1-positive cells in different retinal layers following IVT injection of the indicated viral vectors. (C) Relative mRNA expression levels of TNF-α, IL-1β, IFN-γ, and IL-6, as measured by ddPCR, in the retina 4 weeks after injection of 2.0 × 10^9^ vg/eye of the specified AAV vectors.

Intravitreal injections of AAV vectors typically elicit stronger immune responses compared to subretinal injections.^21^ Engineered vectors, such as AAV2.7m8, appear to cause increased immune responses compared to their wild-type parent, AAV2, likely due to the higher degree of heterogeneity in vector genomes packaged by AAV2.7m8.^22^ To assess whether AAV2.CPP1 induces immune responses in the retina, we injected intravitreally ssAAV2.CB6.*GFP*, ssAAV2.7m8.CB6.*GFP*, and ssAAV2.CPP1.CB6.*GFP* at a dose of 2.0 × 10^9^ vg/eye in mice. Four weeks post-injection, retinal cross sections were analyzed using immunohistochemistry for IBA1, a marker of microglial cells. Increased IBA1 staining intensity indicates microglial activation, which is predominantly seen in the inner retinal layers. We observed a significantly higher number of IBA1-positive microglia in the retinas of mice treated with ssAAV2.7m8.CB6.*GFP* compared to those treated with ssAAV2.CPP1.CB6.*GFP* or ssAAV2.CB6.*GFP* (Figures 4A and S6). Further quantitative analysis revealed that the inner plexiform layer (IPL) of retinas treated with ssAAV2.7m8.CB6.*GFP* had a substantial increase in IBA1-positive microglia, suggesting that microglial cells had migrated into the IPL. In contrast, this increased immune response was not observed in retinas treated with ssAAV2.CPP1.CB6.*GFP* (Figure 4B), indicating a likely reduced immunogenicity of the AAV2.CPP1 vector.

Activated microglia, along with other cell types, may upregulate proinflammatory cytokines such as tumor necrosis factor-alpha (TNF-α), interleukin-1 beta (IL-1β), interferon-gamma (IFN-γ), and interleukin-6 (IL-6). To explore this possibility, we examined RNA levels for these cytokines using droplet digital PCR (ddPCR) in retinas 4 weeks post-injection of different GFP-encoding vectors. Among these cytokines, IL-6 expression was notably higher in retinas injected with ssAAV2.7m8.CB6.*GFP* compared to both uninjected controls and retinas injected with ssAAV2.CB6.*GFP* and ssAAV2.CPP1.CB6.*GFP* (Figure 4C). This suggests that ssAAV2.7m8.CB6.*GFP* likely induces a robust immune response even 4 weeks post-injection. Interestingly, retinas injected with ssAAV2.CPP1.CB6.*GFP* did not show elevated IL-6 levels, indicating a lower degree of immunogenicity associated with the AAV2.CPP1 vector.

### Reduced HS binding affinity of AAV2.CPP1

**Figure 5:**
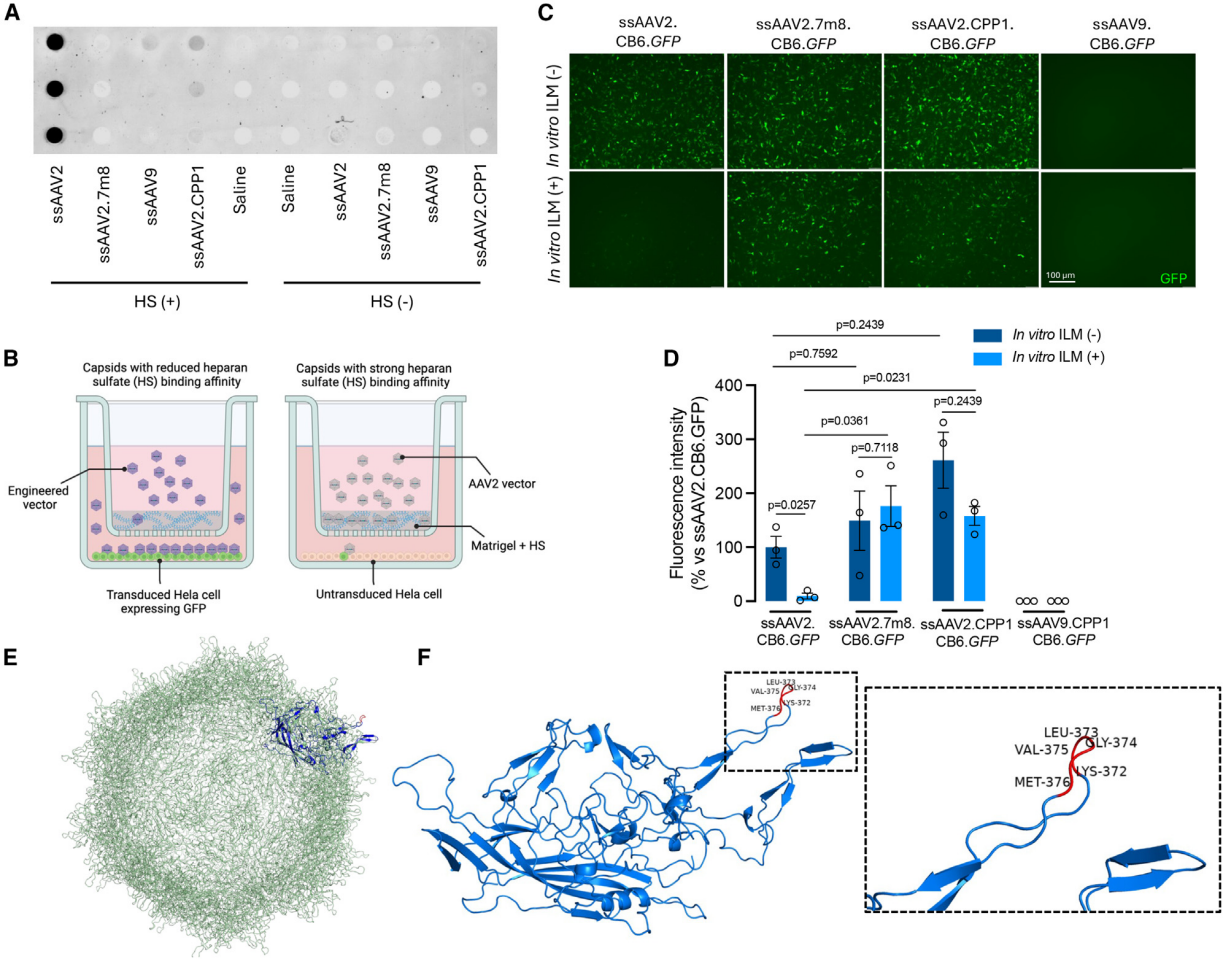
Heparan sulfate binding affinity of AAV2 and AAV2.7m8 vectors (A) Dot blot analysis showing that the AAV2 capsid strongly binds to heparan sulfate (HS), while the AAV2.7m8 capsid shows reduced HS binding. (B) Illustration of the Matrigel-HS-based Transwell model used to differentiate capsids with strong HS binding from those with weak HS binding. (C) The Transwell-based model distinguishes the HS binding affinities of AAV2, AAV2.7m8 and AAV2.CPP1 capsids. AAV9 capsids were added as a negative control. (D) Quantitative analysis of GFP expression indicates that the Matrigel-HS-based Transwell model can effectively distinguish between AAV2, AAV2.7m8, and AAV2.CPP1 capsids, based on their different affinities for HS binding. (E and F) Molecular model of AAV2 containing the KLGVM insertion (highlighted in red) at amino acid N587. The interaction between the inserted loop and other surface loops of the capsid is hypothesized to contribute to the novel properties observed with this vector.

AAV2 is known to utilize HSPG as its primary receptor for binding to host cells, with subsequent cell entry facilitated by proteinous co-receptors. Studies have shown that modifying the AAV2 capsid can enhance photoreceptor transduction via intravitreal injection by reducing its HS binding affinity, thereby enabling more vectors to cross the ILM, as demonstrated by AAV2.7m8.^8^ To investigate the mechanism behind the enhanced retinal transduction of AAV2.CPP1, we assessed the HS binding affinity of ssAAV2.CB6.*GFP*, ssAAV2.7m8.CB6.*GFP*, and ssAAV2.CPP1.CB6.*GFP*. Dot blot analysis using anti-HS antibody to detect vector-HS interactions revealed that AAV2 exhibited strong HS binding, while both AAV2.7m8 and AAV2.CPP1 showed significantly reduced binding to HS (Figure 5A). This reduced HS binding in AAV2.CPP1 suggests that CPP1 may facilitate greater vector penetration across the ILM, leading to enhanced retinal cell transduction.

To further test this hypothesis, we developed a Matrigel-HS-based Transwell model to partially mimic ILM by coating culture Transwells with a mixture of Matrigel and HS and seeding HeLa cells, a cell line rich in HSPG, underneath the Transwells. We proposed that AAVs with strong HS binding would be unable to penetrate this *in vitro* model, thus failing to transduce the HeLa cells. Conversely, AAVs with reduced HS binding would pass through the HS-enriched Transwells and transduce the cells, resulting in detectable GFP expression (Figure 5B). Using this system, we compared the transduction profile of ssAAV2.CB6.*GFP*, ssAAV2.7m8.CB6.*GFP*, ssAAV2.CPP1.CB6.*GFP*, and ssAAV9.CB6.*GFP*. After 48 h of infection, we observed that in the absence of *in vitro* model, GFP expression was comparable among ssAAV2.CB6.*GFP*, ssAAV2.7m8.CB6.*GFP*, and ssAAV2.CPP1.CB6.*GFP*, except for cells transduced by ssAAV9.CB6.GFP, which is known for poor transduction with cells enriched with HSPG. In the presence of the Matrigel-HS-based Transwell model, GFP expression in ssAAV2.CB6.*GFP*-treated cells was significantly reduced, whereas cells treated with ssAAV2.7m8.CB6.*GFP* or ssAAV2.CPP1.CB6.*GFP* maintained GFP expression (Figure 5C). Quantitative analysis (Figure 5D) confirmed these observations, showing a clear distinction in GFP expression levels among the vectors in this *in vitro* model. Importantly, these findings indicate that the CPP1 vector, like 7m8, exhibits reduced HS binding affinity.

We also modeled the AAV2.CPP1 capsid structure by superimposing it onto the AAV2 capsid (Figures 5E and 5F). Notably, the KLGVM peptide insertion disrupted the VR-VIII region, which forms the top of the second highest of the three protrusions at the three-fold axis of symmetry.^23^^,^^24^ This alteration potentially affects the spacing between R585 and R588 and effectively interrupts the HSPG binding motif.

## Discussion

Currently, most AAV-based retinal gene therapies rely on subretinal injections to target photoreceptors and RPE cells, as this approach provides localized, efficient transduction of these critical cells.^25^ However, subretinal injections are invasive and can transduce only a limited retinal area near the injection site. In contrast, intravitreal injections are less invasive and offer the potential for broader retinal coverage but predominantly transduce inner retinal cells, such as ganglion cells, which are not the primary targets for most IRDs.^26^ To address these limitations, early studies focused on replacing tyrosine residues to phenylalanine in AAV2 capsids to reduce ubiquitination, allowing more AAV particles to escape degradation.^27^^,^^28^ More recently, directed evolution has emerged as a primary engineering approach, modifying AAV2 capsids by inserting random 7-mer peptides.^8^^,^^29^ These strategies aim to enhance the vector’s ability to transduce outer retinal cells via intravitreal injection. Given the established ability of CPPs to transport large molecular cargo across cell membranes via direct penetration,^30^^,^^31^ we hypothesized that grafting CPPs into AAV2 capsids could enhance their efficiency in transducing outer retinal cells. Using a photoreceptor-specific, transcription-dependent screening platform,^17^ we identified an AAV2 variant with a 5-mer peptide (KLGVM) insertion, named AAV2.CPP1, following two rounds of screening in mice. This variant demonstrated robust pan-retinal transduction and reduced immune response when delivered via intravitreal injection in mice.

KLGVM is a modified version of KLPVM, a highly effective CPP derived from the Bax-binding domain of Ku70 found in multiple species.^32^ KLPVM shows high cell-entry efficiency, similar to other well-established CPPs like TAT and polyarginine (R8). Its mechanism of entry largely bypasses traditional pinocytosis and endocytosis, as evidenced by minimal inhibition from endocytic inhibitors, except for a partial effect of cytochalasin D.^32^ Moreover, KLPVM does not rely on proteoglycans for cellular entry, and it demonstrates low cytotoxicity even at high concentrations. When fused to Cre recombinase, KLPVM effectively delivers functional proteins into cells, inducing GFP expression. Thus, its derivative, KLGVM, likely shares similar capabilities for intracellular delivery. The pan-retinal transduction observed with AAV2.CPP1 suggests that this vector potentially bypasses the proteoglycan-mediated pathways and instead relies on direct membrane penetration, similar to the mechanism used by KLPVM. In contrast, AAV2.7m8 displayed more localized transduction, particularly around blood vessels, which is likely due to the thinner ILM in these regions, reducing the trapping by HSPGs. In addition, retinas treated with AAV2.CPP1 showed increased GFP mRNA expression compared to those treated with AAV2 and AAV2.7m8, despite similar viral genome levels among all vectors, suggesting potential enhanced cellular entry by AAV2.CPP1. It is not surprising that applying evolutionary pressure based on transgene mRNA levels results in vectors with enhanced RNA expression.^33^ One study demonstrated that the VP1 domain of AAV8 recruits transcription-promoting host factors to vector genomes in both mouse and human cells, leading to increased chromatin accessibility and histone methylation.^34^ Several studies have demonstrated capsid-mediated transcriptional regulation associated with various sites on the AAV capsid, highlighting the multifaceted role of the capsid in transgene transcription.^33^^,^^35^^,^^36^^,^^37^ Future experiments investigating the interaction of AAV2.CPP1 capsids with the intracellular expression machinery could provide further insights into this phenomenon.

One of the challenges in retinal gene therapy is the role of HS binding in limiting AAV2 vectors to the ILM.^7^ While HS binding helps sequester AAVs from the vitreous to the ILM, it may also prevent broader retinal cell transduction. Reducing HS binding, as seen with AAV2.7m8, allows for greater penetration beyond the ILM.^8^ Similarly, two other engineered AAV2 variants, AAV2.GL and AAV2.NN, which enable photoreceptor transduction via intravitreal injection, also exhibit reduced HS binding affinity due to the disruption of canonical HSPG binding at R585 and R588. However, a new binding motif forms through arginine of the inserted peptide and two alanine residues, enhancing receptor interaction. Structural changes and potential deamidation at N587 further contribute to reduced heparin affinity and improved potency of these engineered AAV capsids.^38^ AAV2.CPP1 also showed reduced HS binding, as confirmed by HS binding affinity assays. This finding further supports the hypothesis that reduced HS binding enhances AAV vector penetration.

Despite the promising increase in transgene expression across all retinal layers, including photoreceptors, with AAV2.CPP1 via intravitreal injection, this screening and validation was conducted only in mice, and the results have not yet been confirmed in larger animal models, such as NHPs. Cross-species barriers remain a significant challenge in AAV capsid engineering. For example, AAV.PHP.B and AAV.PHP.eB, which showed a dramatic increase in brain cell transduction in mice via systemic injection, did not replicate this success in NHPs due to species-specific receptor interactions, such as with Ly6a in C57BL mice.^39^^,^^40^ Even AAV2.7m8, which showed effective pan-retinal transduction in mice, achieved transduction only in the macula and peripheral retina of NHPs, where the ILM is thinner. During the AAV library panning process in mice, the disproportionate enrichment of certain CPP variants in each selection round may lead to biases. Dominant variants in a given round might not reflect true enrichment scores, as their proportion in subsequent rounds could skew results. This issue could be mitigated by using synthesized variants with proportional evenness, especially in highly diverse libraries with millions of variants.^17^^,^^41^ Although we observed reduced inflammation and microglia activation with AAV2.CPP1 compared to AAV2.7m8, the long-term effects of immune responses remain unclear. Previous studies have indicated that inflammation induced by AAV2.7m8 is likely due to the packaging of heterogeneous vector genomes, leading to microglial activation.^22^ Investigating the AAV genome homogeneity of AAV2.CPP1 is essential to fully understand its safety profile. Finally, while we hypothesize that KLGVM, as a derivative of KLPVM, enables cell entry through direct membrane penetration, it remains unclear whether engineering KLGVM into the AAV2 capsid affects this entry mechanism. Further studies are needed to confirm whether the incorporation of KLGVM retains the same cell-penetrating characteristics as KLPVM when used in the context of an AAV capsid.

## Materials and methods

### Vector construction

Library backbone vectors containing an AAV capsid expression cassette driven by either the GRK1 or the CB6 promoter were constructed using the Gibson assembly method. For the first-round screening, the backbone plasmid pAAV-Cap2-sc1 included inverted terminal repeats (ITRs) and a partial Rep2 C-terminal sequence, which is crucial for capsid gene splicing and protein assembly. A full-length cap2 gene was inserted between residues N587 and R588 with a unique AflII restriction site for CPP library cloning. For the second-round screening, the backbone plasmid pAAV-Cap2-sc2 also contained ITRs and the partial Rep2 C-terminal sequence, but with a cap2 fragment that featured a unique AflII site for cloning the CPP sequences recovered from the retina. Complete sequences of both constructs are available in the supplemental information. A plasmid, pRep2-3stop, was constructed to exclusively express the Rep protein by introducing three stop codons in the Cap2 gene, thereby preventing the expression of the VP1/2/3 proteins.

### Plasmid library, AAV library construction, and virus production

A double-stranded CPP library was synthesized by GenScript, containing overlapping sequences of the Cap2 gene at the 5′ (5′-CCAACCTCCAGAGAGGCAAC-3′) and 3′ (3′-TCTGCGGTAGCTGCTTGTCT-5′) ends for Gibson assembly, to create the plasmid library for the first round of screening. The library was cloned into the backbone plasmid pAAV-Cap2-sc1 using the standard Gibson assembly method. The pAAV-Cap2-sc1 plasmid (2 μg) was digested with AflII enzyme and purified using a DNA and gel purification kit (D4008, Zymo Research, USA). In a 20-μL Gibson assembly reaction, the purified linear pAAV-Cap2-sc1 was combined with the synthesized double-stranded CPP library inserts (backbone/inserts = 1/5 ratio) in the Gibson Assembly Master Mix (10 μL) and water. The resulting plasmid library was precipitated using isopropanol and concentrated in 5 μL of water. The library (<100 ng/μL in 1 μL) was electroporated into ElectroMAX DH10B cells (18290015, Invitrogen, USA), following the manufacturer’s instructions. Electroporated cells were pooled and cultured in 500 mL of TB medium overnight. The resulting plasmid library was extracted and purified. For the second round of screening, pAAV-Cap2-sc2 was digested with AflII and purified in the same manner. The recovered CPP sequences from the retina (after the first round) were cloned into the pAAV-Cap2-sc2 using Gibson assembly to generate the second plasmid library.

AAV library production was conducted in HEK293 cells. Calcium phosphate transfection was performed using 1.5 mg of pAd-DeltaF6 (containing adenovirus helper genes), 1.0 mg of pRep2-3stop, and 100 μg of either the first or the second plasmid library in 10 roller bottles. Cells and culture medium were harvested 72 h post-transfection by scraping, and the cells were pelleted via low-speed centrifugation. The cells were lysed by undergoing three freeze-thaw cycles, and the supernatant was precipitated on ice using 1:0.3 volume of 40% polyethylene glycol solution, followed by centrifugation. The lysate and supernatant were pooled and fractionated through four rounds of iodixanol gradient purification. Buffer exchange was performed on Amicon-100 columns (Millipore) using phosphate-buffered saline (PBS). Final viral preparations were analyzed by ddPCR using a poly(A)-specific primer/probe set (Table S1), and the samples were tested via silver staining of PAGE gels. Full sequences of all the plasmids used in AAV library production can be found in the supplemental information.

### Characterization of AAV particles by high-resolution TEM

The morphology of negatively stained AAV virions was analyzed using TEM at the Core Electron Microscopy Facility of the University of Massachusetts Chan Medical School, following established protocols.^20^ Briefly, 5 μL of the AAV preparation was applied to a Formvar-coated grid and allowed to adhere for 30 s. Excess liquid was removed, and the sample was negatively stained by sequentially flowing six drops of 1% uranyl acetate over the grid, enhancing contrast and fixing the virus particles. After drying in a controlled humidity chamber, the grid was examined using TEM, and images were recorded. The resulting visualization prominently displayed AAV empty particles as characteristic donut-like shapes, formed by the accumulation of uranyl acetate stain in the capsid dimples. To assess the full/empty capsid ratio, six fields of images were analyzed, and the numbers of full and completely or partially empty virions were counted. The average counts from these fields were used to calculate the full/empty capsid ratio.

### Animals

Adult male C57BL/6J mice (5–8 weeks of age) were purchased from The Jackson Laboratory and received intravitreal injections. The mice were maintained on a 12-h light/dark cycle at a temperature of 70°F–74°F and a humidity level of 35%–46%. They were provided with a standard chow diet (ISO-pro 300 irradiated diet, #5P76). All animal procedures in this study were approved by the UMass Chan Medical School Animal Care and Use Committee.

### Library screening by *in vivo* selection

AAV libraries were intravitreally injected into the eyes of C57BL/6J mice (*n* = 10) at a dose of 2.0 × 10^9^ vg/eye. Four weeks post-injection, the animals were euthanized, and the retina/RPE complexes were harvested, pooled, snap-frozen in liquid nitrogen, and stored at −80°C. Total retinal RNA was extracted using the DNA/RNA extraction kit (SKU 47700, Norgen Biotek). mRNA was further purified from the total RNA using the Dynabeads mRNA Purification Kit (#61006, Invitrogen). Reverse transcription was performed using 400 ng of purified mRNA and the gene-specific Cap2-RT primer (Table S1) with the SuperScript IV First-Strand Synthesis Kit (Life Technologies). To amplify the Cap sequence with CPP insertions, a nested PCR approach was used. In the first round of PCR, the target sequence was amplified using the initial set of primers (Nes-1F and Nes-1R) in multiple reactions containing 2 μL of cDNA, 25 μL of Q5 HotStart High-Fidelity 2× Master Mix (NEB), and a total reaction volume of 50 μL for 10 cycles. In the second round, amplification was performed with the second set of primers (Nes-2F and Nes-2R) in multiple reactions using 1 μL of amplicon purified and concentrated from the first-round PCR, 12.5 μL of Q5 2× Master Mix, and a total reaction volume of 25 μL for 20 cycles. The resulting amplicons were purified and concentrated for NGS preparation, using 100 ng of input amplicons in 15 cycles of amplification. The final purified amplicon was ready for NGS sequencing.

### AAV vector production and intravitreal administration in mice

The leading CPP variant enriched in the retina was identified through NGS and subsequently cloned into the Rep/Cap2 plasmid at position N587. Plasmids encoding the Rep and AAV2 (Rep/Cap2), 7m8 (Rep/7m8), and AAV2-CPP (Rep/CPP) capsids were used to produce single-stranded or self-complementary AAV vectors encoding GFP or H2BGFP under the control of the CB6 or GRK1 promoters. This was achieved using the same method as for AAV library production, involving the co-transfection of HEK293 cells. A total of 1.5 mg of pAd-DeltaF6; 150 μg of pssAAV-CB6-GFP, pssAAV-GRK1-H2BGFP (a generous gift of Dr. Claudio Punzo, UMass Chan), or pscAAV-CB6-GFP; and 1.5 mg of Rep/Cap, Rep/7m8, or Rep/CPP was transfected into cells in 10 roller bottles. Seventy-two hours post-transfection, the cells were harvested, lysed, and subjected to iodixanol gradient purification and buffer exchange, following the same protocol used for the AAV libraries. The final titer was confirmed by ddPCR using a GFP probe.

For *in vivo* experiments, viral vectors were intravitreally injected into the eyes of mice at different doses. The injections were administered using glass micropipettes (Clunbury Scientific LLC), introduced through the corneal limbus, with fast green dye added at a concentration of 0.1% to visualize the injection site. Each mouse received 1 μL of vector. Twenty-eight days post-injection, mice were anesthetized for fluorescence fundus imaging and subsequently euthanized for retinal harvesting for immunohistochemistry and molecular analysis.

### NGS and bioinformatic analysis

Amplicon libraries for NGS were prepared by performing 15 cycles of PCR amplification on 100 ng of the second round of nested PCR amplicons, using Q5 High-Fidelity DNA polymerase (NEB) with the NGS-1F and NGS-1R primers. The amplicon libraries were then purified using the Zymo Gel DNA Recovery Kit (Zymo Research). The final libraries were quantified with the Qubit dsDNA HS kit (Life Technology) before being submitted for sequencing at the Massachusetts General Hospital (MGH) DNA core facility. FASTQ files generated from the sequencing were processed using Geneious Prime software, which enabled quantitative and qualitative assessment of the peptide sequence inserts.

### Fluorescence fundus imaging and immunohistochemistry

To perform fundus imaging, mice were first administered one drop each of phenylephrine and tropicamide to dilate the pupils. The mice were then anesthetized via intraperitoneal injection of a ketamine/xylazine mixture (100 and 10 mg/kg). A mouse was positioned on a fundus scope platform, and a heating pad was used to maintain lens clarity by keeping the mouse warm. The platform was adjusted to position the camera directly over the eye. Appropriate wavelengths were selected to capture focused images of the retina, and images were acquired with the Micron IV system from the Phoenix Technology Group (Lakewood, CO, USA).

The eye cups, after removing the cornea, were fixed overnight at 4°C in 4% paraformaldehyde, followed by dehydration in 30% sucrose at 4°C overnight before embedding. Cryosections, 12 μm thick, were prepared as previously described.^42^ The sections were analyzed using a Leica DM6 Thunder microscope equipped with a 16-bit monochrome camera. After post-fixation and rinsing in PBS, the sections were blocked and incubated with antibodies overnight at 4°C. The following antibodies were used: chicken anti-EGFP (ab13970; 1:1,000; Abcam), rabbit anti-IBA1 (019-19741; 1:300; Wako), and fluorescein peanut agglutinin lectin (PNA) (FL1071; 1:1,000; Vector Laboratories). All images were visualized with a Leica DM6 Thunder microscope with a 16-bit monochrome camera. Images were processed by LAS X Life Science Microscope Software.

### Quantification of photoreceptor transduction

Quantification of photoreceptor transduction in retinas treated with vectors encoding cytosolic GFP under the CB6 promoter was performed by manually counting GFP-positive photoreceptor segments. For each group of injected mice, four rectangular regions of equal area surrounding the optic nerve head were selected from a single retinal section per eye. GFP-positive segments within these selected areas were manually counted. In addition, the total number of photoreceptor nuclei in the outer nuclear layer within the same areas was determined using the Imaris software package (8.2).^43^ Photoreceptor transduction efficiency was then calculated as the average per eye and per group by determining the ratio of GFP-positive photoreceptors to the total number of photoreceptors. For retinas treated with vectors encoding nuclear GFP under the GRK1 promoter, GFP-positive photoreceptor nuclei were manually counted across the entire retinal section. The total number of photoreceptors and the photoreceptor transduction efficiency were calculated as described previously.

### Quantitative analysis of viral genome and transcripts

Snap-frozen retinas were processed for total DNA and RNA extraction using the DNA/RNA Extraction Kit (SKU 47700, Norgen Biotek). RNA was reverse transcribed into cDNA using the cDNA Reverse Transcription Kit (#4374966; Thermo Fisher Scientific). Vector DNA and cDNA were quantified using duplex TaqMan ddPCR assays targeting EGFP (assay ID Mr00660654_cn; Thermo Fisher Scientific) and Tfrc genomic sequences (#4458367; Thermo Fisher Scientific) or EGFP and Gusb cDNA (#4448490; Thermo Fisher Scientific). The ddPCR was performed on a QX200 system (Bio-Rad), and data analysis was conducted using QuantaSoft software (Bio-Rad).

### HS binding affinity assay and dot-plot analysis

For the HS affinity assay, 500 μL of diluted AAV vector containing 1.0 × 10^10^ vg was incubated with or without 2 μL of HS solution (100 μg/μL) for 1 h at room temperature, with shaking at 500 rpm. After incubation, 100 μL of the mixture was loaded onto a nitrocellulose membrane using a blot filtration system. The membrane was quickly rinsed once with 0.5% PBST and then blocked with blocking solution for 1 h at room temperature. HS antibody (clone F58-10E4; 1:200; Amsbio) was used for incubation overnight at 4°C. The membrane was washed three times with 0.5% PBST for 5 min each, followed by incubation with the secondary antibody (IRDye 680; 1:5,000; LI-COR). A final quick rinse with 0.5% PBST was performed before imaging.

### The Matrigel-HS-based Transwell model

A Matrigel-HS-based Transwell model was established to assess the HS binding affinity of AAV2 variants. HeLa cells were seeded at a density of 1.0 × 10^5^ cells per well in a 24-well plate the day before AAV infection. The culture inserts (CLS3470; 6.5-mm Transwell with 0.4-μm pore polyester membrane insert; Corning) were coated with Matrigel (5 μg/cm^2^; CLS356231; Corning) mixed with HS solution (300 μg HS in 70 μL of Matrigel solution; S5992; Selleck Chemicals) and incubated at 37°C for 5 h. After incubation, the remaining solution in the inserts was removed, and the inserts were mounted into the wells containing the seeded cells. AAV vectors encoding the *GFP* gene were then added to the inserts at a multiplicity of infection (MOI) of 100,000 in 100 μL of culture medium mixed with Ad5 helper virus (MOI of 100).^44^ GFP expression was assessed through fluorescence microscopy to evaluate transduction efficiency 2 days post-infection.

### Molecular modeling

The monomeric structure of the VP1 capsid (amino acid residues 219–740) was predicted using AlphaFold 3.0 (https://alphafoldserver.com). The prediction was based on the AAV2 VP1 reference model (PDB: 6IH9) with a resolution of 2.8 Å. Default automated settings were applied to build the VP3 monomer. The predicted VP3 monomer was then aligned with the wild-type AAV2 VP3 monomer using PyMOL software. Capsid reconstruction was carried out using the Viper server (https://viperdb.org/Oligomer_Generator.php).

### Statistical analysis

Data were analyzed using GraphPad Prism version 10 (GraphPad Software) and are reported as means ± standard deviation (SD). Depending on the experimental setup, statistical comparisons were performed using one-way ANOVA followed by Tukey’s multiple comparisons test. Statistical significance was defined as an adjusted *p* value of less than 0.05.

## Data availability

All data generated in this study are included in this published article and in the supplemental information. All these data will be made available upon request.

## Acknowledgments

The authors are grateful to the Viral Vector Core at UMass Chan for producing the AAV vectors used in this study. J.-H.W. is supported by the Graduate Education Fund of the American Australian Association and Australian Vision Research. G.G. is supported by 10.13039/100000002NIH grants (R01NS076991-01, P01HL131471-05, R01AI121135, UG3HL147367-01, R01HL097088, R01HL152723-02, U19AI149646-01, and UH3HL147367-04). Some figures were created with Biorender.com.

## Author contributions

Conceptualization, J.-H.W. and G.G.; methodology, J.-H.W., M.C., H.L., and G.G.; formal analysis, J.-H.W., M.C., H.L., P.G., D.J.G., J.M., and S.-Y.C.; resources, G.G.; data curation, J.-H.W., M.C., and H.L.; writing – original draft, J.-H.W.; writing – review & editing, J.-H.W., M.C., H.L., D.J.G., J.X., C.P., P.W.L.T., and G.G.; visualization, J.-H.W. and M.C.; supervision, G.G.; project administration, J.-H.W. and G.G.; funding acquisition, G.G. All authors have read and approved the article.

## Declaration of interests

G.G. is a scientific co-founder of Voyager Therapeutics, Adrenas Therapeutics, and Aspa Therapeutics and holds equity in these companies. G.G. is an inventor on patents with potential royalties licensed to Voyager Therapeutics, Aspa Therapeutics, and other biopharmaceutical companies. D.J.G. is a scientific co-founder of Aspa Therapeutics. D.J.G. is an inventor on patents with potential royalties licensed to Aspa Therapeutics and other biopharmaceutical companies.

## Supplemental information

Document S1. Figures S1–S6 and Table S1

Document S2. Article plus supplemental information

## References

[1] Wang D., Tai P.W.L., Gao G. (2019). Adeno-associated virus vector as a platform for gene therapy delivery. Nat. Rev. Drug Discov..

[2] Wang J.-H., Gessler D.J., Zhan W., Gallagher T.L., Gao G. (2024). Adeno-associated virus as a delivery vector for gene therapy of human diseases. Signal Transduct. Targeted Ther..

[3] Wang J.-H., Zhan W., Gallagher T.L., Gao G. (2024). Recombinant Adeno-Associated Virus as a Delivery Platform for Ocular Gene Therapy: A Comprehensive Review. Mol. Ther..

[4] Maguire A.M., Bennett J., Aleman E.M., Leroy B.P., Aleman T.S. (2021). Clinical Perspective: Treating RPE65-Associated Retinal Dystrophy. Mol. Ther..

[5] Duncan J.L., Pierce E.A., Laster A.M., Daiger S.P., Birch D.G., Ash J.D., Iannaccone A., Flannery J.G., Sahel J.A., Zack D.J. (2018). Inherited Retinal Degenerations: Current Landscape and Knowledge Gaps. Transl. Vis. Sci. Technol..

[6] Li C., Samulski R.J. (2020). Engineering adeno-associated virus vectors for gene therapy. Nat. Rev. Genet..

[7] Woodard K.T., Liang K.J., Bennett W.C., Samulski R.J. (2016). Heparan Sulfate Binding Promotes Accumulation of Intravitreally Delivered Adeno-associated Viral Vectors at the Retina for Enhanced Transduction but Weakly Influences Tropism. J. Virol..

[8] Dalkara D., Byrne L.C., Klimczak R.R., Visel M., Yin L., Merigan W.H., Flannery J.G., Schaffer D.V. (2013). In vivo-directed evolution of a new adeno-associated virus for therapeutic outer retinal gene delivery from the vitreous. Sci. Transl. Med..

[9] Zhang K.Y., Johnson T.V. (2021). The internal limiting membrane: Roles in retinal development and implications for emerging ocular therapies. Exp. Eye Res..

[10] Herce H.D., Garcia A.E. (2007). Cell Penetrating Peptides: How Do They Do It?. J. Biol. Phys..

[11] Nhàn N.T.T., Maidana D.E., Yamada K.H. (2023). Ocular Delivery of Therapeutic Agents by Cell-Penetrating Peptides. Cells.

[12] de Cogan F., Hill L.J., Lynch A., Morgan-Warren P.J., Lechner J., Berwick M.R., Peacock A.F.A., Chen M., Scott R.A.H., Xu H. (2017). Topical Delivery of Anti-VEGF Drugs to the Ocular Posterior Segment Using Cell-Penetrating Peptides. Investig. Ophthalmol. Vis. Sci..

[13] Yao Y., Wang J., Liu Y., Qu Y., Wang K., Zhang Y., Chang Y., Yang Z., Wan J., Liu J. (2022). Variants of the adeno-associated virus serotype 9 with enhanced penetration of the blood-brain barrier in rodents and primates. Nat. Biomed. Eng..

[14] Deverman B.E., Pravdo P.L., Simpson B.P., Kumar S.R., Chan K.Y., Banerjee A., Wu W.-L., Yang B., Huber N., Pasca S.P., Gradinaru V. (2016). Cre-dependent selection yields AAV variants for widespread gene transfer to the adult brain. Nat. Biotechnol..

[15] Chan K.Y., Jang M.J., Yoo B.B., Greenbaum A., Ravi N., Wu W.-L., Sánchez-Guardado L., Lois C., Mazmanian S.K., Deverman B.E., Gradinaru V. (2017). Engineered AAVs for efficient noninvasive gene delivery to the central and peripheral nervous systems. Nat. Neurosci..

[16] Agrawal P., Bhalla S., Usmani S.S., Singh S., Chaudhary K., Raghava G.P.S., Gautam A. (2016). CPPsite 2.0: a repository of experimentally validated cell-penetrating peptides. Nucleic Acids Res..

[17] Nonnenmacher M., Wang W., Child M.A., Ren X.Q., Huang C., Ren A.Z., Tocci J., Chen Q., Bittner K., Tyson K. (2021). Rapid evolution of blood-brain-barrier-penetrating AAV capsids by RNA-driven biopanning. Mol. Ther. Methods Clin. Dev..

[18] Büning H., Srivastava A. (2019). Capsid Modifications for Targeting and Improving the Efficacy of AAV Vectors. Mol. Ther. Methods Clin. Dev..

[19] Nonnenmacher M., van Bakel H., Hajjar R.J., Weber T. (2015). High Capsid–Genome Correlation Facilitates Creation of AAV Libraries for Directed Evolution. Mol. Ther..

[20] Gao K., Li M., Zhong L., Su Q., Li J., Li S., He R., Zhang Y., Hendricks G., Wang J., Gao G. (2014). Empty virions in AAV8 vector preparations reduce transduction efficiency and may cause total viral particle dose-limiting side effects. Mol. Ther. Methods Clin. Dev..

[21] Chan Y.K., Dick A.D., Hall S.M., Langmann T., Scribner C.L., Mansfield B.C., Ocular Gene Therapy Inflammation Working Group (2021). Inflammation in Viral Vector-Mediated Ocular Gene Therapy: A Review and Report From a Workshop Hosted by the Foundation Fighting Blindness, 9/2020. Transl. Vis. Sci. Technol..

[22] Cui M., Su Q., Yip M., McGowan J., Punzo C., Gao G., Tai P.W.L. (2024). The AAV2.7m8 capsid packages a higher degree of heterogeneous vector genomes than AAV2. Gene Ther..

[23] Tseng Y.-S., Agbandje-McKenna M. (2014). Mapping the AAV Capsid Host Antibody Response toward the Development of Second Generation Gene Delivery Vectors. Front. Immunol..

[24] Perabo L., Goldnau D., White K., Endell J., Boucas J., Humme S., Work L.M., Janicki H., Hallek M., Baker A.H., Büning H. (2006). Heparan sulfate proteoglycan binding properties of adeno-associated virus retargeting mutants and consequences for their *in vivo* tropism. J. Virol..

[25] Trapani I., Auricchio A. (2018). Seeing the Light after 25 Years of Retinal Gene Therapy. Trends Mol. Med..

[26] Xue K., Groppe M., Salvetti A.P., MacLaren R.E. (2017). Technique of retinal gene therapy: delivery of viral vector into the subretinal space. Eye.

[27] Zhong L., Li B., Jayandharan G., Mah C.S., Govindasamy L., Agbandje-McKenna M., Herzog R.W., Weigel-Van Aken K.A., Hobbs J.A., Zolotukhin S. (2008). Tyrosine-phosphorylation of AAV2 vectors and its consequences on viral intracellular trafficking and transgene expression. Virology.

[28] Petrs-Silva H., Dinculescu A., Li Q., Deng W.-T., Pang J.J., Min S.-H., Chiodo V., Neeley A.W., Govindasamy L., Bennett A. (2011). Novel Properties of Tyrosine-mutant AAV2 Vectors in the Mouse Retina. Mol. Ther..

[29] Byrne L.C., Day T.P., Visel M., Strazzeri J.A., Fortuny C., Dalkara D., Merigan W.H., Schaffer D.V., Flannery J.G. (2020). In vivo–directed evolution of adeno-associated virus in the primate retina. JCI Insight.

[30] Ruseska I., Zimmer A. (2020). Internalization mechanisms of cell-penetrating peptides. Beilstein J. Nanotechnol..

[31] Palm-Apergi C., Lönn P., Dowdy S.F. (2012). Do Cell-Penetrating Peptides Actually “Penetrate” Cellular Membranes?. Mol. Ther..

[32] Gomez J.A., Chen J., Ngo J., Hajkova D., Yeh I.-J., Gama V., Miyagi M., Matsuyama S. (2010). Cell-Penetrating Penta-Peptides (CPP5s): Measurement of Cell Entry and Protein-Transduction Activity. Pharmaceuticals.

[33] Hoffman J.A., Denton N., Sims J.J., Meggersee R., Zhang Z., Olagbegi K., Wilson J.M. (2024). Modulation of AAV9 Galactose Binding Yields Novel Gene Therapy Vectors and Predicts Cross-Species Differences in Glycan Avidity. Hum. Gene Ther..

[34] Loeb E.J., Havlik P.L., Elmore Z.C., Rosales A., Fergione S.M., Gonzalez T.J., Smith T.J., Benkert A.R., Fiflis D.N., Asokan A. (2024). Capsid-mediated control of adeno-associated viral transcription determines host range. Cell Rep..

[35] Chuapoco M.R., Flytzanis N.C., Goeden N., Christopher Octeau J., Roxas K.M., Chan K.Y., Scherrer J., Winchester J., Blackburn R.J., Campos L.J. (2023). Adeno-associated viral vectors for functional intravenous gene transfer throughout the non-human primate brain. Nat. Nanotechnol..

[36] Salganik M., Aydemir F., Nam H.-J., McKenna R., Agbandje-McKenna M., Muzyczka N. (2014). Adeno-Associated Virus Capsid Proteins May Play a Role in Transcription and Second-Strand Synthesis of Recombinant Genomes. J. Virol..

[37] Powell S.K., Samulski R.J., McCown T.J. (2020). AAV Capsid-Promoter Interactions Determine CNS Cell-Selective Gene Expression In Vivo. Mol. Ther..

[38] Pavlou M., Schön C., Occelli L.M., Rossi A., Meumann N., Boyd R.F., Bartoe J.T., Siedlecki J., Gerhardt M.J., Babutzka S. (2021). Novel AAV capsids for intravitreal gene therapy of photoreceptor disorders. EMBO Mol. Med..

[39] Huang Q., Chan K.Y., Tobey I.G., Chan Y.A., Poterba T., Boutros C.L., Balazs A.B., Daneman R., Bloom J.M., Seed C., Deverman B.E. (2019). Delivering genes across the blood-brain barrier: LY6A, a novel cellular receptor for AAV-PHP.B capsids. PLoS One.

[40] Hordeaux J., Yuan Y., Clark P.M., Wang Q., Martino R.A., Sims J.J., Bell P., Raymond A., Stanford W.L., Wilson J.M. (2019). The GPI-Linked Protein LY6A Drives AAV-PHP.B Transport across the Blood-Brain Barrier. Mol. Ther..

[41] Tabebordbar M., Lagerborg K.A., Stanton A., King E.M., Ye S., Tellez L., Krunnfusz A., Tavakoli S., Widrick J.J., Messemer K.A. (2021). Directed evolution of a family of AAV capsid variants enabling potent muscle-directed gene delivery across species. Cell.

[42] Cheng S.-Y., Cipi J., Ma S., Hafler B.P., Kanadia R.N., Brush R.S., Agbaga M.-P., Punzo C. (2020). Altered photoreceptor metabolism in mouse causes late stage age-related macular degeneration-like pathologies. Proc. Natl. Acad. Sci. USA.

[43] Petit L., Ma S., Cheng S.-Y., Gao G., Punzo C. (2017). Rod Outer Segment Development Influences AAV-Mediated Photoreceptor Transduction After Subretinal Injection. Hum. Gene Ther..

[44] Chang H., Du A., Jiang J., Ren L., Liu N., Zhou X., Liang J., Gao G., Wang D. (2023). Non-canonical amino acid incorporation into AAV5 capsid enhances lung transduction in mice. Mol. Ther. Methods Clin. Dev..

