# Supplementary material for: Cell-penetrating peptide-grafted AAV2 capsids for improved retinal delivery via intravitreal injection
Source: Mol Ther Methods Clin Dev. 2025 Feb 3;33(1):101426. doi: 10.1016/j.omtm.2025.101426 (PMC11872077; doi:10.1016/j.omtm.2025.101426)
Supplement: Document S1. Figures S1–S6 and Table S1 [file mmc1.pdf]

OMTM, Volume *33*

Supplemental information

Cell-penetrating peptide-grafted AAV2 capsids for improved retinal delivery via intravitreal injection

Jiang-Hui Wang, Mengtian Cui, Hao Liu, Peiyi Guo, Jackson McGowan, Shun-Yun Cheng, Dominic J. Gessler, Jun Xie, Claudio Punzo, Phillip W.L. Tai, and Guangping Gao

Table S1. Primers used in the study.

Primer name	Primer sequence (5' to 3')
Cap2-RT	TACGAGTCAGGTATCTGGTGCCAA
Nes-1F	GGAGCCTCGAACGACAATCACTAC
Nes-1R	ACAGACTTGTTGTAGTTGGAAGTGT
Nes-2F	ACGTGACTGGCAAAGACTCATCAA
Nes-2R	CTGACCTGTCCCGTGGAGT
NGS-1F	ATCTTTGGGAAGCAAGGCTCAGAG
NGS-1R	CCTTGTGTGTTGACATCTGCGGTA

Nucleotide sequence of CPP1 (KLGVM): AAGCTGGGCGTGATG

Synthesized CPP library oligo DNA sequence:
5' CCAACCTCCAGAGAGGCAACNNKNNKNNK……AGACAAGCAGCTACCGCAGA 3'

Vector sequences:
pAAV-Cap2-sc1 (6418bp)

Features:
11–140: 5' ITR
214–505: GRK1
523–731: AAV2 Rep C-terminal sequence
1027–3225: Cap2 fragment sequence
2788–2792: Inserted digestion site of AflII
3263–3389: Rabbit globin polyA
3607–3478: 3' ITR
4370–5227: Ampicillin resistance

CTTAATTAGGCTGCGCGCTCGCTCGCTCACTGAGGCCGCCCGGGCAAAGCCCGGGCGTCGGGCGACCT
TTGGTCGCCCGGCCTCAGTGAGCGAGCGAGCGCGCAGAGAGGGAGTGGCCAACTCCATCACTAGGGG
TTCCTTGTAGTTAATGATTAACCCGCCATGCTACTTATCTACCAGGGTAATGGGACGCGTGATCCTCTA
GAACTATAGGGGCCCCAGAAGCCTGGTGGTTGTTTGTCCTTCTCAGGGGAAAAGTGAGGCGGCCCCTT
GGAGGAAGGGGCCGGGCAGAATGATCTAATCGGATTCCAAGCAGCTCAGGGGATTGTCTTTTTCTAGC
ACCTTCTTGCCACTCCTAAGCGTCCTCCGTGACCCCGGCTGGGATTTAGCCTGGTGCTGTGTCAGCCCC
GGTCTCCCAGGGGCTTCCCAGTGGTCCCCAGGAACCCTCGACAGGGCCCGGTCTCTCTCGTCCAGCAA
GGGCAGGGACGGGCCACAGGCCAAGGGCCGGGAGCAAGCTGCTAGCGGTCACCAAGCAGGAAGTCA
AAGACTTTTTCCGGTGGGCAAAGGATCACGTGGTTGAGGTGGAGCATGAATTCTACGTCAAAAAGGGT
GGAGCCAAGAAAAGACCCGCCCCCAGTGACGCAGATATAAGTGAGCCCAAACGGGTGCGCGAGTCAG
TTGCGCAGCCATCGACGTCAGACGCGGAAGCTTCGATCAACTACGCGGACAGGTACCAAAACAAATG
TTCTCGTCACGTGGGCATGAATCTGATGCTGTTTCCCTGCAGACAATGCGAGAGAATGAATCAGAATT
CAAATATCTGCTTCACTCACGGACAGAAAGACTGTTTAGAGTGCTTTCCCGTGTCAGAATCTCAACCCG
TTTCTGTCGTCAAAAAGGCGTATCAGAAACTGTGCTACATTCATCATATCATGGGAAAGGTGCCAGAC
GCTTGCACTGCCTGCGATCTGGTCAATGTGGATTTGGATGACTGCATCTTTGAACAATAAATGATTTAA
ATCAGGTATGGCTGCCGATGGTTATCTTCCAGATTGGCTCGAGGACACTCTCTCTGAAGGAATAAGAC
AGTGGTGGAAGCTCAAACCTGGCCCACCACCACCAAAGCCCGCAGAGCGGCATAAGGACGACAGCAG
GGGTCTTGTGCTTCCTGGGTACAAGTACCTCGGACCCTTCAACGGACTCGACAAGGGAGAGCCGGTCA

ACGAGGCAGACGCCGCGGCCCTCGAGCACGACAAAGCCTACGACCGGCAGCTCGACAGCGGAGACAA
CCCGTACCTCAAGTACAACCACGCCGACGCGGAGTTTCAGGAGCGCCTTAAAGAAGATACGTCTTTTG
GGGGCAACCTCGGACGAGCAGTCTTCCAGGCGAAAAAGAGGGTTCTTGAACCTCTGGGCCTGGTTGAG
GAACCTGTTAAGACGGCTCCGGGAAAAAAGAGGCCGGTAGAGCACTCTCCTGTGGAGCCAGACTCCT
CCTCGGGAACCGGAAAGGCGGGCCAGCAGCCTGCAAGAAAAAGATTGAATTTTGGTCAGACTGGAGA
CGCAGACTCAGTACCTGACCCCCAGCCTCTCGGACAGCCACCAGCAGCCCCCTCTGGTCTGGGAACTA
ATACGATGGCTACAGGCAGTGGCGCACCAATGGCAGACAATAACGAGGGCGCCGACGGAGTGGGTAA
TTCCTCGGGAAATTGGCATTGCGATTCCACATGGATGGGCGACAGAGTCATCACCACCAGCACCCGAA
CCTGGGCCCTGCCCACCTACAACAACCACCTCTACAAACAAATTTCCAGCCAATCAGGAGCCTCGAAC
GACAATCACTACTTTGGCTACAGCACCCCTTGGGGGTATTTTGACTTCAACAGATTCCACTGCCACTTT
TCACCACGTGACTGGCAAAGACTCATCAACAACAACTGGGGATTCCGACCCAAGAGACTCAACTTCAA
GCTCTTTAACATTCAAGTCAAAGAGGTCACGCAGAATGACGGTACGACGACGATTGCCAATAACCTTA
CCAGCACGGTTCAGGTGTTTACTGACTCGGAGTACCAGCTCCCGTACGTCCTCGGCTCGGCGCATCAA
GGATGCCTCCCGCCGTTCCCAGCAGACGTCTTCATGGTGCCACAGTATGGATACCTCACCCTGAACAA
CGGGAGTCAGGCAGTAGGACGCTCTTCATTTTACTGCCTGGAGTACTTTCCTTCTCAGATGCTGCGTAC
CGGAAACAACTTTACCTTCAGCTACACTTTTGAGGACGTTCCTTTCCACAGCAGCTACGCTCACAGCCA
GAGTCTGGACCGTCTCATGAATCCTCTCATCGACCAGTACCTGTATTACTTGAGCAGAACAAACACTCC
AAGTGGAACCACCACGCAGTCAAGGCTTCAGTTTTCTCAGGCCGGAGCGAGTGACATTCGGGACCAGT
CTAGGAACTGGCTTCCTGGACCCTGTTACCGCCAGCAGCGAGTATCAAAGACATCTGCGGATAACAAC
AACAGTGAATACTCGTGGACTGGAGCTACCAAGTACCACCTCAATGGCAGAGACTCTCTGGTGAATCC
GGGCCCGGCCATGGCAAGCCACAAGGACGATGAAGAAAAGTTTTTCCTCAGAGCGGGGTTCTCATCT
TTGGGAAGCAAGGCTCAGAGAAAACAAATGTGGACATTGAAAAGGTCATGATTACAGACGAAGAGGA
AATCAGGACAACCAATCCCGTGGCTACGGAGCAGTATGGTTCTGTATCTACCAACCTCCAGAGAGGCA
ACTTAAGAGACAAGCAGCTACCGCAGATGTCAACACACAAGGCGTTCTTCCAGGCATGGTCTGGCAGG
ACAGAGATGTGTACCTTCAGGGGCCCATCTGGGCAAAGATTCCACACACGGACGGACATTTTCACCCC
TCTCCCCTCATGGGTGGATTCGGACTTAAACACCCTCCTCCACAGATTCTCATCAAGAACACCCCGGTA
CCTGCGAATCCTTCGACCACCTTCAGTGCGGCAAAGTTTGCTTCCTTCATCACACAGTACTCCACGGGA
CAGGTCAGCGTGGAGATCGAGTGGGAGCTGCAGAAGGAAAACAGCAAACGCTGGAATCCCGAAATTC
AGTACACTTCCAACTACAACAAGTCTGTTAATGTGGACTTTACTGTGGACACTAATGGCGTGTATTCAG
AGCCTCGCCCCATTGGCACCAGATACCTGACTCGTAATCTGTAATAAGCGGCCGCCTCGAGTGATCCG
ATCTTTTTCCCTCTGCCAAAAATTATGGGGACATCATGAAGCCCCTTGAGCATCTGACTTCTGGCTAAT
AAAGGAAATTTATTTTCATTGCAATAGTGTGTTGGAATTTTTTGTGTCTCTCACTCGGAAGCAATTCGT
TGATCTGAATTTCGACCACCCATAATACCCATTACCCTGGTAGATAAGTAGCATGGCGGGTTAATCATT
AACTACAAGGAACCCCTAGTGATGGAGTTGGCCACTCCCTCTCTGCGCGCTCGCTCGCTCACTGAGGC
CGGGCGACCAAAGGTCGCCCGACGCCCGGGCTTTGCCCGGGCGGCCTCAGTGAGCGAGCGAGCGCGC
AGCCTTAATTAACCTAATTCACTGGCCGTCGTTTTACAACGTCGTGACTGGGAAAACCCTGGCGTTACC
CAACTTAATCGCCTTGCAGCACATCCCCCTTTCGCCAGCTGGCGTAATAGCGAAGAGGCCCGCACCGA
TCGCCCTTCCCAACAGTTGCGCAGCCTGAATGGCGAATGGGACGCGCCCTGTAGCGGCGCATTAAGCG
CGGCGGGTGTGGTGGTTACGCGCAGCGTGACCGCTACACTTGCCAGCGCCCTAGCGCCCGCTCCTTTC
GCTTTCTTCCCTTCCTTTCTCGCCACGTTCGCCGGCTTTCCCCGTCAAGCTCTAAATCGGGGGCTCCCTT
TAGGGTTCCGATTTAGTGCTTTACGGCACCTCGACCCCAAAAAACTTGATTAGGGTGATGGTTCACGTA
GTGGGCCATCGCCCTGATAGACGGTTTTTCGCCCTTTGACGTTGGAGTCCACGTTCTTTAATAGTGGAC
TCTTGTTCCAAACTGGAACAACACTCAACCCTATCTCGGTCTATTCTTTTGATTTATAAGGGATTTTGCC
GATTTCGGCCTATTGGTTAAAAAATGAGCTGATTTAACAAAAATTTAACGCGAATTTTAACAAAATAT
TAACGCTTACAATTTAGGTGGCACTTTTCGGGGAAATGTGCGCGGAACCCCTATTTGTTTATTTTTCTA
AATACATTCAAATATGTATCCGCTCATGAGACAATAACCCTGATAAATGCTTCAATAATATTGAAAAA
GGAAGAGTATGAGTATTCAACATTTCCGTGTCGCCCTTATTCCCTTTTTTGCGGCATTTTGCCTTCCTGT
TTTTGCTCACCCAGAAACGCTGGTGAAAGTAAAAGATGCTGAAGATCAGTTGGGTGCACGAGTGGGTT
ACATCGAACTGGATCTCAACAGCGGTAAGATCCTTGAGAGTTTTCGCCCCGAAGAACGTTTTCCAATG
ATGAGCACTTTTAAAGTTCTGCTATGTGGCGCGGTATTATCCCGTATTGACGCCGGGCAAGAGCAACT
CGGTCGCCGCATACACTATTCTCAGAATGACTTGGTTGAGTACTCACCAGTCACAGAAAAGCATCTTA
CGGATGGCATGACAGTAAGAGAATTATGCAGTGCTGCCATAACCATGAGTGATAACACTGCGGCCAA
CTTACTTCTGACAACGATCGGAGGACCGAAGGAGCTAACCGCTTTTTTGCACAACATGGGGGATCATG
TAACTCGCCTTGATCGTTGGGAACCGGAGCTGAATGAAGCCATACCAAACGACGAGCGTGACACCAC
GATGCCTGTAGCAATGGCAACAACGTTGCGCAAACTATTAACTGGCGAACTACTTACTCTAGCTTCCC
GGCAACAATTAATAGACTGGATGGAGGCGGATAAAGTTGCAGGACCACTTCTGCGCTCGGCCCTTCCG

GCTGGCTGGTTTATTGCTGATAAATCTGGAGCCGGTGAGCGTGGGTCTCGCGGTATCATTGCAGCACT
GGGGCCAGATGGTAAGCCCTCCCGTATCGTAGTTATCTACACGACGGGGAGTCAGGCAACTATGGATG
AACGAAATAGACAGATCGCTGAGATAGGTGCCTCACTGATTAAGCATTGGTAACTGTCAGACCAAGTT
TACTCATATATACTTTAGATTGATTTAAAACTTCATTTTTAATTTAAAAGGATCTAGGTGAAGATCCTTT
TTGATAATCTCATGACCAAAATCCCTTAACGTGAGTTTTCGTTCCACTGAGCGTCAGACCCCGTAGAAA
AGATCAAAGGATCTTCTTGAGATCCTTTTTTTCTGCGCGTAATCTGCTGCTTGCAAACAAAAAAACCAC
CGCTACCAGCGGTGGTTTGTTTGCCGGATCAAGAGCTACCAACTCTTTTTCCGAAGGTAACTGGCTTCA
GCAGAGCGCAGATACCAAATACTGTTCTTCTAGTGTAGCCGTAGTTAGGCCACCACTTCAAGAACTCT
GTAGCACCGCCTACATACCTCGCTCTGCTAATCCTGTTACCAGTGGCTGCTGCCAGTGGCGATAAGTCG
TGTCTTACCGGGTTGGACTCAAGACGATAGTTACCGGATAAGGCGCAGCGGTCGGGCTGAACGGGGG
GTTCGTGCACACAGCCCAGCTTGGAGCGAACGACCTACACCGAACTGAGATACCTACAGCGTGAGCTA
TGAGAAAGCGCCACGCTTCCCGAAGGGAGAAAGGCGGACAGGTATCCGGTAAGCGGCAGGGTCGGAA
CAGGAGAGCGCACGAGGGAGCTTCCAGGGGGAAACGCCTGGTATCTTTATAGTCCTGTCGGGTTTCGC
CACCTCTGACTTGAGCGTCGATTTTTGTGATGCTCGTCAGGGGGGCGGAGCCTATGGAAAAACGCCAG
CAACGCGGCCTTTTTACGGTTCCTGGCCTTTTGCTGGCCTTTTGCTCACATGTTCTTTCCTGCGTTATCC
CCTGATTCTGTGGATAACCGTATTACCGCCTTTGAGTGAGCTGATACCGCTCGCCGCAGCCGAACGAC
CGAGCGCAGCGAGTCAGTGAGCGAGGAAGCGGAAGAGCGCCCAATACGCAAACCGCCTCTCCCCGCG
CGTTGGCCGATTCATTAATGCAGCTGGCACGACAGGTTTCCCGACTGGAAAGCGGGCAGTGAGCGCAA
CGCAATTAATGTGAGTTAGCTCACTCATTAGGCACCCCAGGCTTTACACTTTATGCTTCCGGCTCGTAT
GTTGTGTGGAATTGTGAGCGGATAACAATTTCACACAGGAAACAGCTATGACCATGATTACGCCAGAT
TTAATTAAGGC

pAAV-Cap2-sc2 (6373bp)

Features:
11–140: 5’ ITR
214–505: GRK1
523–731: AAV2 Rep C-terminal sequence
1027–3180: Cap2 fragment sequence
2770–2774: Inserted digestion site of AflII
3218–3344: Rabbit globin polyA
3562–3433: 3’ ITR
4325–5182: Ampicillin resistance

CTTAATTAGGCTGCGCGCTCGCTCGCTCACTGAGGCCGCCCGGGCAAAGCCCGGGCGTCGGGCGACCT
TTGGTCGCCCGGCCTCAGTGAGCGAGCGAGCGCGCAGAGAGGGAGTGGCCAACTCCATCACTAGGGG
TTCCTTGTAGTTAATGATTAACCCGCCATGCTACTTATCTACCAGGGTAATGGGACGCGTGATCCTCTA
GAACTATAGGGGCCCCAGAAGCCTGGTGGTTGTTTGTCCTTCTCAGGGGAAAAGTGAGGCGGCCCCTT
GGAGGAAGGGGCCGGGCAGAATGATCTAATCGGATTCCAAGCAGCTCAGGGGATTGTCTTTTTCTAGC
ACCTTCTTGCCACTCCTAAGCGTCCTCCGTGACCCCGGCTGGGATTTAGCCTGGTGCTGTGTCAGCCCC
GGTCTCCCAGGGGCTTCCCAGTGGTCCCCAGGAACCCTCGACAGGGCCCGGTCTCTCTCGTCCAGCAA
GGGCAGGGACGGGCCACAGGCCAAGGGCCGGGAGCAAGCTGCTAGCGGTCACCAAGCAGGAAGTCA
AAGACTTTTTCCGGTGGGCAAAGGATCACGTGGTTGAGGTGGAGCATGAATTCTACGTCAAAAAGGGT
GGAGCCAAGAAAAGACCCGCCCCCAGTGACGCAGATATAAGTGAGCCCAAACGGGTGCGCGAGTCAG
TTGCGCAGCCATCGACGTCAGACGCGGAAGCTTCGATCAACTACGCGGACAGGTACCAAAACAAATG
TTCTCGTCACGTGGGCATGAATCTGATGCTGTTTCCCTGCAGACAATGCGAGAGAATGAATCAGAATT
CAAATATCTGCTTCACTCACGGACAGAAAGACTGTTTAGAGTGCTTTCCCGTGTCAGAATCTCAACCCG
TTTCTGTCGTCAAAAAGGCGTATCAGAAACTGTGCTACATTCATCATATCATGGGAAAGGTGCCAGAC
GCTTGCACTGCCTGCGATCTGGTCAATGTGGATTTGGATGACTGCATCTTTGAACAATAAATGATTTAA
ATCAGGTATGGCTGCCGATGGTTATCTTCCAGATTGGCTCGAGGACACTCTCTCTGAAGGAATAAGAC
AGTGGTGGAAGCTCAAACCTGGCCCACCACCACCAAAGCCCGCAGAGCGGCATAAGGACGACAGCAG
GGGTCTTGTGCTTCCTGGGTACAAGTACCTCGGACCCTTCAACGGACTCGACAAGGGAGAGCCGGTCA
ACGAGGCAGACGCCGCGGCCCTCGAGCACGACAAAGCCTACGACCGGCAGCTCGACAGCGGAGACAA

CCCGTACCTCAAGTACAACCACGCCGACGCGGAGTTTCAGGAGCGCCTTAAAGAAGATACGTCTTTTG
GGGGCAACCTCGGACGAGCAGTCTTCCAGGCGAAAAAGAGGGTTCTTGAACCTCTGGGCCTGGTTGAG
GAACCTGTTAAGACGGCTCCGGGAAAAAAGAGGCCGGTAGAGCACTCTCCTGTGGAGCCAGACTCCT
CCTCGGGAACCGGAAAGGCGGGCCAGCAGCCTGCAAGAAAAAGATTGAATTTTGGTCAGACTGGAGA
CGCAGACTCAGTACCTGACCCCCAGCCTCTCGGACAGCCACCAGCAGCCCCCTCTGGTCTGGGAACTA
ATACGATGGCTACAGGCAGTGGCGCACCAATGGCAGACAATAACGAGGGCGCCGACGGAGTGGGTAA
TTCCTCGGGAAATTGGCATTGCGATTCCACATGGATGGGCGACAGAGTCATCACCACCAGCACCCGAA
CCTGGGCCCTGCCCACCTACAACAACCACCTCTACAAACAAATTTCCAGCCAATCAGGAGCCTCGAAC
GACAATCACTACTTTGGCTACAGCACCCCTTGGGGGTATTTTGACTTCAACAGATTCCACTGCCACTTT
TCACCACGTGACTGGCAAAGACTCATCAACAACAACTGGGGATTCCGACCCAAGAGACTCAACTTCAA
GCTCTTTAACATTCAAGTCAAAGAGGTCACGCAGAATGACGGTACGACGACGATTGCCAATAACCTTA
CCAGCACGGTTCAGGTGTTTACTGACTCGGAGTACCAGCTCCCGTACGTCCTCGGCTCGGCGCATCAA
GGATGCCTCCCGCCGTTCCCAGCAGACGTCTTCATGGTGCCACAGTATGGATACCTCACCCTGAACAA
CGGGAGTCAGGCAGTAGGACGCTCTTCATTTTACTGCCTGGAGTACTTTCCTTCTCAGATGCTGCGTAC
CGGAAACAACTTTACCTTCAGCTACACTTTTGAGGACGTTCCTTTCCACAGCAGCTACGCTCACAGCCA
GAGTCTGGACCGTCTCATGAATCCTCTCATCGACCAGTACCTGTATTACTTGAGCAGAACAAACACTCC
AAGTGGAACCACCACGCAGTCAAGGCTTCAGTTTTCTCAGGCCGGAGCGAGTGACATTCGGGACCAGT
CTAGGAACTGGCTTCCTGGACCCTGTTACCGCCAGCAGCGAGTATCAAAGACATCTGCGGATAACAAC
AACAGTGAATACTCGTGGACTGGAGCTACCAAGTACCACCTCAATGGCAGAGACTCTCTGGTGAATCC
GGGCCCGGCCATGGCAAGCCACAAGGACGATGAAGAAAAGTTTTTCCTCAGAGCGGGGTTCTCATCT
TTGGGAAGCAAGGCTCAGAGAAAACAAATGTGGACATTGAAAAGGTCATGATTACAGACGAAGAGGA
AATCAGGACAACCAATCCCGTGGCTACGGAGCAGTATGGTTCTGTATCTACCTTAAGACACAAGGCGT
TCTTCCAGGCATGGTCTGGCAGGACAGAGATGTGTACCTTCAGGGGCCCATCTGGGCAAAGATTCCAC
ACACGGACGGACATTTTCACCCCTCTCCCCTCATGGGTGGATTCGGACTTAAACACCCTCCTCCACAGA
TTCTCATCAAGAACACCCCGGTACCTGCGAATCCTTCGACCACCTTCAGTGCGGCAAAGTTTGCTTCCT
TCATCACACAGTACTCCACGGGACAGGTCAGCGTGGAGATCGAGTGGGAGCTGCAGAAGGAAAACAG
CAAACGCTGGAATCCCGAAATTCAGTACACTTCCAACTACAACAAGTCTGTTAATGTGGACTTTACTGT
GGACACTAATGGCGTGTATTCAGAGCCTCGCCCCATTGGCACCAGATACCTGACTCGTAATCTGTAAT
AAGCGGCCGCCTCGAGTGATCCGATCTTTTTCCCTCTGCCAAAAATTATGGGGACATCATGAAGCCCCT
TGAGCATCTGACTTCTGGCTAATAAAGGAAATTTATTTTCATTGCAATAGTGTGTTGGAATTTTTTGTG
TCTCTCACTCGGAAGCAATTCGTTGATCTGAATTTCGACCACCCATAATACCCATTACCCTGGTAGATA
AGTAGCATGGCGGGTTAATCATTAACTACAAGGAACCCCTAGTGATGGAGTTGGCCACTCCCTCTCTG
CGCGCTCGCTCGCTCACTGAGGCCGGGCGACCAAAGGTCGCCCGACGCCCGGGCTTTGCCCGGGCGGC
CTCAGTGAGCGAGCGAGCGCGCAGCCTTAATTAACCTAATTCACTGGCCGTCGTTTTACAACGTCGTG
ACTGGGAAAACCCTGGCGTTACCCAACTTAATCGCCTTGCAGCACATCCCCCTTTCGCCAGCTGGCGTA
ATAGCGAAGAGGCCCGCACCGATCGCCCTTCCCAACAGTTGCGCAGCCTGAATGGCGAATGGGACGC
GCCCTGTAGCGGCGCATTAAGCGCGGCGGGTGTGGTGGTTACGCGCAGCGTGACCGCTACACTTGCCA
GCGCCCTAGCGCCCGCTCCTTTCGCTTTCTTCCCTTCCTTTCTCGCCACGTTCGCCGGCTTTCCCCGTCA
AGCTCTAAATCGGGGGCTCCCTTTAGGGTTCCGATTTAGTGCTTTACGGCACCTCGACCCCAAAAAACT
TGATTAGGGTGATGGTTCACGTAGTGGGCCATCGCCCTGATAGACGGTTTTTCGCCCTTTGACGTTGGA
GTCCACGTTCTTTAATAGTGGACTCTTGTTCCAAACTGGAACAACACTCAACCCTATCTCGGTCTATTC
TTTTGATTTATAAGGGATTTTGCCGATTTCGGCCTATTGGTTAAAAAATGAGCTGATTTAACAAAAATT
TAACGCGAATTTTAACAAAATATTAACGCTTACAATTTAGGTGGCACTTTTCGGGGAAATGTGCGCGG
AACCCCTATTTGTTTATTTTTCTAAATACATTCAAATATGTATCCGCTCATGAGACAATAACCCTGATA
AATGCTTCAATAATATTGAAAAAGGAAGAGTATGAGTATTCAACATTTCCGTGTCGCCCTTATTCCCTT
TTTTGCGGCATTTTGCCTTCCTGTTTTTGCTCACCCAGAAACGCTGGTGAAAGTAAAAGATGCTGAAGA
TCAGTTGGGTGCACGAGTGGGTTACATCGAACTGGATCTCAACAGCGGTAAGATCCTTGAGAGTTTTC
GCCCCGAAGAACGTTTTCCAATGATGAGCACTTTTAAAGTTCTGCTATGTGGCGCGGTATTATCCCGTA
TTGACGCCGGGCAAGAGCAACTCGGTCGCCGCATACACTATTCTCAGAATGACTTGGTTGAGTACTCA
CCAGTCACAGAAAAGCATCTTACGGATGGCATGACAGTAAGAGAATTATGCAGTGCTGCCATAACCAT
GAGTGATAACACTGCGGCCAACTTACTTCTGACAACGATCGGAGGACCGAAGGAGCTAACCGCTTTTT
TGCACAACATGGGGGATCATGTAACTCGCCTTGATCGTTGGGAACCGGAGCTGAATGAAGCCATACCA
AACGACGAGCGTGACACCACGATGCCTGTAGCAATGGCAACAACGTTGCGCAAACTATTAACTGGCG
AACTACTTACTCTAGCTTCCCGGCAACAATTAATAGACTGGATGGAGGCGGATAAAGTTGCAGGACCA
CTTCTGCGCTCGGCCCTTCCGGCTGGCTGGTTTATTGCTGATAAATCTGGAGCCGGTGAGCGTGGGTCT
CGCGGTATCATTGCAGCACTGGGGCCAGATGGTAAGCCCTCCCGTATCGTAGTTATCTACACGACGGG

GAGTCAGGCAACTATGGATGAACGAAATAGACAGATCGCTGAGATAGGTGCCTCACTGATTAAGCATT
GGTAACTGTCAGACCAAGTTTACTCATATATACTTTAGATTGATTTAAAACTTCATTTTTAATTTAAAA
GGATCTAGGTGAAGATCCTTTTTGATAATCTCATGACCAAAATCCCTTAACGTGAGTTTTCGTTCCACT
GAGCGTCAGACCCCGTAGAAAAGATCAAAGGATCTTCTTGAGATCCTTTTTTTCTGCGCGTAATCTGCT
GCTTGCAAACAAAAAAACCACCGCTACCAGCGGTGGTTTGTTTGCCGGATCAAGAGCTACCAACTCTT
TTTCCGAAGGTAACTGGCTTCAGCAGAGCGCAGATACCAAATACTGTTCTTCTAGTGTAGCCGTAGTTA
GGCCACCACTTCAAGAACTCTGTAGCACCGCCTACATACCTCGCTCTGCTAATCCTGTTACCAGTGGCT
GCTGCCAGTGGCGATAAGTCGTGTCTTACCGGGTTGGACTCAAGACGATAGTTACCGGATAAGGCGCA
GCGGTCGGGCTGAACGGGGGGTTCGTGCACACAGCCCAGCTTGGAGCGAACGACCTACACCGAACTG
AGATACCTACAGCGTGAGCTATGAGAAAGCGCCACGCTTCCCGAAGGGAGAAAGGCGGACAGGTATC
CGGTAAGCGGCAGGGTCGGAACAGGAGAGCGCACGAGGGAGCTTCCAGGGGGAAACGCCTGGTATCT
TTATAGTCCTGTCGGGTTTCGCCACCTCTGACTTGAGCGTCGATTTTTGTGATGCTCGTCAGGGGGGCG
GAGCCTATGGAAAAACGCCAGCAACGCGGCCTTTTTACGGTTCCTGGCCTTTTGCTGGCCTTTTGCTCA
CATGTTCTTTCCTGCGTTATCCCCTGATTCTGTGGATAACCGTATTACCGCCTTTGAGTGAGCTGATACC
GCTCGCCGCAGCCGAACGACCGAGCGCAGCGAGTCAGTGAGCGAGGAAGCGGAAGAGCGCCCAATAC
GCAAACCGCCTCTCCCCGCGCGTTGGCCGATTCATTAATGCAGCTGGCACGACAGGTTTCCCGACTGG
AAAGCGGGCAGTGAGCGCAACGCAATTAATGTGAGTTAGCTCACTCATTAGGCACCCCAGGCTTTACA
CTTTATGCTTCCGGCTCGTATGTTGTGTGGAATTGTGAGCGGATAACAATTTCACACAGGAAACAGCTA
TGACCATGATTACGCCAGATTTAATTAAGGC

pRep2-3stop

Features:
151–750: stuffer
775–2717: Rep2 fragment
2178–4468: Cap2 fragment
3144–3146, 3144–3146, 3363–3365: stop codon
5797–6654: Ampicillin resistance

CTCTAGAGGTCCTGTATTAGAGGTCACGTGAGTGTTTTGCGACATTTTGCGACACCATGTGGTCACGCT
GGGTATTTAAGCCCGAGTGAGCACGCAGGGTCTCCATTTTGAAGCGGGAGGTTTGAACGCGCAGCCGC
CAAGCCGAATTCTGCAGATATCCCCGAGTCCTTCAATGCTATCATTCCCTTTGATATTGGACCATATGC
ATAGTACCGAGAAACTAGTGCGAAGTAGTGATCAGGTATTGCTGTTAGATATCCCCGAGTCCTTCAAT
GCTATCATTCTCTTTGATATTGGACCATATGCATAGTACCGAGAAACTAGTGCGAAGTAGTGATCAGG
TATTGCTGTTAGATATCCCCGAGTCCTTCAATGCTATCATTCCCTTTGATATTGGACCATATGCATAGTA
CCGAGAAACTAGTGCGAAGTAGTGATCAGGTATTGCTGTTAGATATCCCCGAGTCCTTCAATGCTATC
ATTCTCTTTGATATTGGACCATATGCATAGTACCGAGAAACTAGTGCGAAGTAGTGATCAGGTATTGCT
GTTAGATATCCCCGAGTCCTTCAATGCTATCATTCCCTTTGATATTGGACCATATGCATAGTACCGAGA
AACTAGTGCGAAGTAGTGATCAGGTATTGCTGTTAGATATCCCCGAGTCCTTCAATGCTATCATTTCCT
TTGATATTGGATCATATGCATAGTACCGAGAAACTAGTGCGAAGTAGTGATCAGGTATTGCTGTTAAG
GATCCATCACACTGGCGGCCGCTCGAGGGGAGCTCGCAGGGTCTCCATTTTGAAGCGGGAGGTTTGAA
CGCGCAGCCGCCATGCCGGGGTTTTACGAGATTGTGATTAAGGTCCCCAGCGACCTTGACGAGCATCT
GCCCGGCATTTCTGACAGCTTTGTGAACTGGGTGGCCGAGAAGGAATGGGAGTTGCCGCCAGATTCTG
ACATGGATCTGAATCTGATTGAGCAGGCACCCCTGACCGTGGCCGAGAAGCTGCAGCGCGACTTTCTG
ACGGAATGGCGCCGTGTGAGTAAGGCCCCGGAGGCTCTTTTCTTTGTGCAATTTGAGAAGGGAGAGAG
CTACTTCCACATGCACGTGCTCGTGGAAACCACCGGGGTGAAATCCATGGTTTTGGGACGTTTCCTGA
GTCAGATTCGCGAAAAACTGATTCAGAGAATTTACCGCGGGATCGAGCCGACTTTGCCAAACTGGTTC
GCGGTCACAAAGACCAGAAATGGCGCCGGAGGCGGGAACAAGGTGGTGGATGAGTGCTACATCCCCA
ATTACTTGCTCCCCAAAACCCAGCCTGAGCTCCAGTGGGCGTGGACTAATATGGAACAGTATTTAAGC
GCCTGTTTGAATCTCACGGAGCGTAAACGGTTGGTGGCGCAGCATCTGACGCACGTGTCGCAGACGCA
GGAGCAGAACAAAGAGAATCAGAATCCCAATTCTGATGCGCCGGTGATCAGATCAAAAACTTCAGCC
AGGTACATGGAGCTGGTCGGGTGGCTCGTGGACAAGGGGATTACCTCGGAGAAGCAGTGGATCCAGG
AGGACCAGGCCTCATACATCTCCTTCAATGCGGCCTCCAACTCGCGGTCCCAAATCAAGGCTGCCTTG
GACAATGCGGGAAAGATTATGAGCCTGACTAAAACCGCCCCCGACTACCTGGTGGGCCAGCAGCCCG

TGGAGGACATTTCCAGCAATCGGATTTATAAAATTTTGGAACTAAACGGGTACGATCCCCAATATGCG
GCTTCCGTCTTTCTGGGATGGGCCACGAAAAAGTTCGGCAAGAGGAACACCATCTGGCTGTTTGGGCC
TGCAACTACCGGGAAGACCAACATCGCGGAGGCCATAGCCCACACTGTGCCCTTCTACGGGTGCGTAA
ACTGGACCAATGAGAACTTTCCCTTCAACGACTGTGTCGACAAGATGGTGATCTGGTGGGAGGAGGGG
AAGATGACCGCCAAGGTCGTGGAGTCGGCCAAAGCCATTCTCGGAGGAAGCAAGGTGCGCGTGGACC
AGAAATGCAAGTCCTCGGCCCAGATAGACCCGACTCCCGTGATCGTCACCTCCAACACCAACATGTGC
GCCGTGATTGACGGGAACTCAACGACCTTCGAACACCAGCAGCCGTTGCAAGACCGGATGTTCAAATT
TGAACTCACCCGCCGTCTGGATCATGACTTTGGGAAGGTCACCAAGCAGGAAGTCAAAGACTTTTTCC
GGTGGGCAAAGGATCACGTGGTTGAGGTGGAGCATGAATTCTACGTCAAAAAGGGTGGAGCCAAGAA
AAGACCCGCCCCCAGTGACGCAGATATAAGTGAGCCCAAACGGGTGCGCGAGTCAGTTGCGCAGCCA
TCGACGTCAGACGCGGAAGCTTCGATCAACTACGCAGACAGGTACCAAAACAAATGTTCTCGTCACGT
GGGCATGAATCTGATGCTGTTTCCCTGCAGACAATGCGAGAGAATGAATCAGAATTCAAATATCTGCT
TCACTCACGGACAGAAAGACTGTTTAGAGTGCTTTCCCGTGTCAGAATCTCAACCCGTTTCTGTCGTCA
AAAAGGCGTATCAGAAACTGTGCTACATTCATCATATCATGGGAAAGGTGCCAGACGCTTGCACTGCC
TGCGATCTGGTCAATGTGGATTTGGATGACTGCATCTTTGAACAATAAATGATTTAAATCAGGTATGGC
TGCCGATGGTTAGCTTCCAGATTGGCTCGAGGACACTCTCTCTGAAGGAATAAGACAGTGGTGGAAGC
TCAAACCTGGCCCACCACCACCAAAGCCCGCAGAGCGGCATAAGGACGACAGCAGGGGTCTTGTGCT
TCCTGGGTACAAGTACCTCGGACCCTTCAACGGACTCGACAAGGGAGAGCCGGTCAACGAGGCAGAC
GCCGCGGCCCTCGAGCACGACAAAGCCTACGACCGGCAGCTCGACAGCGGAGACAACCCGTACCTCA
AGTACAACCACGCCGACGCGGAGTTTCAGGAGCGCCTTAAAGAAGATACGTCTTTTGGGGGCAACCTC
GGACGAGCAGTCTTCCAGGCGAAAAAGAGGGTTCTTGAACCTCTGGGCCTGGTTGAGGAACCTGTTAA
GACGGCTCCGGGAAAATAGAGGCCGGTAGAGCACTCTCCTGTGGAGCCAGACTCCTCCTCGGGAACC
GGAAAGGCGGGCCAGCAGCCTGCAAGAAAAAGATTGAATTTTGGTCAGACTGGAGACGCAGACTCAG
TACCTGACCCCCAGCCTCTCGGACAGCCACCAGCAGCCCCCTCTGGTCTGGGAACTAATACGATGGCT
ACAGGCAGTGGCGCACCAATGGCAGACAATAACTAGGGCGCCGACGGAGTGGGTAATTCCTCGGGAA
ATTGGCATTGCGATTCCACATGGATGGGCGACAGAGTCATCACCACCAGCACCCGAACCTGGGCCCTG
CCCACCTACAACAACCACCTCTACAAACAAATTTCCAGCCAATCAGGAGCCTCGAACGACAATCACTA
CTTTGGCTACAGCACCCCTTGGGGGTATTTTGACTTCAACAGATTCCACTGCCACTTTTCACCACGTGA
CTGGCAAAGACTCATCAACAACAACTGGGGATTCCGACCCAAGAGACTCAACTTCAAGCTCTTTAACA
TTCAAGTCAAAGAGGTCACGCAGAATGACGGTACGACGACGATTGCCAATAACCTTACCAGCACGGTT
CAGGTGTTTACTGACTCGGAGTACCAGCTCCCGTACGTCCTCGGCTCGGCGCATCAAGGATGCCTCCC
GCCGTTCCCAGCAGACGTCTTCATGGTGCCACAGTATGGATACCTCACCCTGAACAACGGGAGTCAGG
CAGTAGGACGCTCTTCATTTTACTGCCTGGAGTACTTTCCTTCTCAGATGCTGCGTACCGGAAACAACT
TTACCTTCAGCTACACTTTTGAGGACGTTCCTTTCCACAGCAGCTACGCTCACAGCCAGAGTCTGGACC
GTCTCATGAATCCTCTCATCGACCAGTACCTGTATTACTTGAGCAGAACAAACACTCCAAGTGGAACC
ACCACGCAGTCAAGGCTTCAGTTTTCTCAGGCCGGAGCGAGTGACATTCGGGACCAGTCTAGGAACTG
GCTTCCTGGACCCTGTTACCGCCAGCAGCGAGTATCAAAGACATCTGCGGATAACAACAACAGTGAAT
ACTCGTGGACTGGAGCTACCAAGTACCACCTCAATGGCAGAGACTCTCTGGTGAATCCGGGCCCGGCC
ATGGCAAGGTCAGCGTGGAGATCGAGTGGGAGCTGCAGAAGGAAAACAGCAAACGCTGGAATCCCGA
AATTCAGTACACTTCCAACTACAACAAGTCTGTTAATGTGGACTTTACTGTGGACACTAATGGCGTGTA
TTCAGAGCCTCGCCCCATTGGCACCAGATACCTGACTCGTAATCTGTAATTGCTTGTTAATCAATAAAC
CGTTTAATTCGTTTCAGTTGAACTTTGGTCTCTGCGTATTTCTTTCTTATCTAGTTTCCATGCTCTAGAGC
GGCCGCCACCGCGGTGGAGCTCCAGCTTTTGTTCCCTTTAGTGAGGGTTAATTGCGCGCTTGGCGTAAT
CATGGTCATAGCTGTTTCCTGTGTGAAATTGTTATCCGCTCACAATTCCACACAACATACGAGCCGGAA
GCATAAAGTGTAAAGCCTGGGGTGCCTAATGAGTGAGCTAACTCACATTAATTGCGTTGCGCTCACTG
CCCGCTTTCCAGTCGGGAAACCTGTCGTGCCAGCTGCATTAATGAATCGGCCAACGCGCGGGGAGAGG
CGGTTTGCGTATTGGGCGCTCTTCCGCTTCCTCGCTCACTGACTCGCTGCGCTCGGTCGTTCGGCTGCG
GCGAGCGGTATCAGCTCACTCAAAGGCGGTAATACGGTTATCCACAGAATCAGGGGATAACGCAGGA
AAGAACATGTGAGCAAAAGGCCAGCAAAAGGCCAGGAACCGTAAAAAGGCCGCGTTGCTGGCGTTTT
TCCATAGGCTCCGCCCCCCTGACGAGCATCACAAAAATCGACGCTCAAGTCAGAGGTGGCGAAACCCG
ACAGGACTATAAAGATACCAGGCGTTTCCCCCTGGAAGCTCCCTCGTGCGCTCTCCTGTTCCGACCCTG
CCGCTTACCGGATACCTGTCCGCCTTTCTCCCTTCGGGAAGCGTGGCGCTTTCTCATAGCTCACGCTGT
AGGTATCTCAGTTCGGTGTAGGTCGTTCGCTCCAAGCTGGGCTGTGTGCACGAACCCCCCGTTCAGCCC
GACCGCTGCGCCTTATCCGGTAACTATCGTCTTGAGTCCAACCCGGTAAGACACGACTTATCGCCACTG
GCAGCAGCCACTGGTAACAGGATTAGCAGAGCGAGGTATGTAGGCGGTGCTACAGAGTTCTTGAAGT
GGTGGCCTAACTACGGCTACACTAGAAGAACAGTATTTGGTATCTGCGCTCTGCTGAAGCCAGTTACC

TTCGGAAAAAGAGTTGGTAGCTCTTGATCCGGCAAACAAACCACCGCTGGTAGCGGTGGTTTTTTTGTT
TGCAAGCAGCAGATTACGCGCAGAAAAAAAGGATCTCAAGAAGATCCTTTGATCTTTTCTACGGGGTC
TGACGCTCAGTGGAACGAAAACTCACGTTAAGGGATTTTGGTCATGAGATTATCAAAAAGGATCTTCA
CCTAGATCCTTTTAAATTAAAAATGAAGTTTTAAATCAATCTAAAGTATATATGAGTAAACTTGGTCTG
ACAGTTACCAATGCTTAATCAGTGAGGCACCTATCTCAGCGATCTGTCTATTTCGTTCATCCATAGTTG
CCTGACTCCCCGTCGTGTAGATAACTACGATACGGGAGGGCTTACCATCTGGCCCCAGTGCTGCAATG
ATACCGCGAGACCCACGCTCACCGGCTCCAGATTTATCAGCAATAAACCAGCCAGCCGGAAGGGCCG
AGCGCAGAAGTGGTCCTGCAACTTTATCCGCCTCCATCCAGTCTATTAATTGTTGCCGGGAAGCTAGA
GTAAGTAGTTCGCCAGTTAATAGTTTGCGCAACGTTGTTGCCATTGCTACAGGCATCGTGGTGTCACGC
TCGTCGTTTGGTATGGCTTCATTCAGCTCCGGTTCCCAACGATCAAGGCGAGTTACATGATCCCCCATG
TTGTGCAAAAAAGCGGTTAGCTCCTTCGGTCCTCCGATCGTTGTCAGAAGTAAGTTGGCCGCAGTGTTA
TCACTCATGGTTATGGCAGCACTGCATAATTCTCTTACTGTCATGCCATCCGTAAGATGCTTTTCTGTG
ACTGGTGAGTACTCAACCAAGTCATTCTGAGAATAGTGTATGCGGCGACCGAGTTGCTCTTGCCCGGC
GTCAATACGGGATAATACCGCGCCACATAGCAGAACTTTAAAAGTGCTCATCATTGGAAAACGTTCTT
CGGGGCGAAAACTCTCAAGGATCTTACCGCTGTTGAGATCCAGTTCGATGTAACCCACTCGTGCACCC
AACTGATCTTCAGCATCTTTTACTTTCACCAGCGTTTCTGGGTGAGCAAAAACAGGAAGGCAAAATGC
CGCAAAAAAGGGAATAAGGGCGACACGGAAATGTTGAATACTCATACTCTTCCTTTTTCAATATTATT
GAAGCATTTATCAGGGTTATTGTCTCATGAGCGGATACATATTTGAATGTATTTAGAAAAATAAACAA
ATAGGGGTTCCGCGCACATTTCCCCGAAAAGTGCCACCTAAATTGTAAGCGTTAATATTTTGTTAAAAT
TCGCGTTAAATTTTTGTTAAATCAGCTCATTTTTTAACCAATAGGCCGAAATCGGCAAAATCCCTTATA
AATCAAAAGAATAGACCGAGATAGGGTTGAGTGTTGTTCCAGTTTGGAACAAGAGTCCACTATTAAAG
AACGTGGACTCCAACGTCAAAGGGCGAAAAACCGTCTATCAGGGCGATGGCCCACTACGTGAACCAT
CACCCTAATCAAGTTTTTTGGGGTCGAGGTGCCGTAAAGCACTAAATCGGAACCCTAAAGGGAGCCCC
CGATTTAGAGCTTGACGGGGAAAGCCGGCGAACGTGGCGAGAAAGGAAGGGAAGAAAGCGAAAGGA
GCGGGCGCTAGGGCGCTGGCAAGTGTAGCGGTCACGCTGCGCGTAACCACCACACCCGCCGCGCTTAA
TGCGCCGCTACAGGGCGCGTCCCATTCGCCATTCAGGCTGCGCAACTGTTGGGAAGGGCGATCGGTGC
GGGCCTCTTCGCTATTACGCCAGCTGGCGAAAGGGGGATGTGCTGCAAGGCGATTAAGTTGGGTAACG
CCAGGGTTTTCCCAGTCACGACGTTGTAAAACGACGGCCAGTGAGCGCGCGTAATACGACTCACTATA
GGGCGAATTGGGTACCGGGCCCCCCCTCGAGGTCGACGGTATGATCCACTAGTAACGGCCGCCAGTGT
GCTGGAATTCGGCTTTGTAGTTAATGATTAACCCGGCATGCTACTTATCTACGTAGCCATG

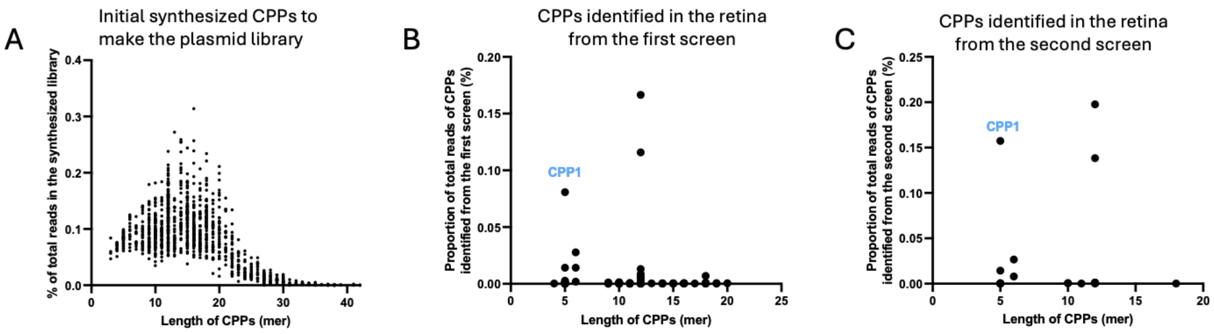

Figure S1. The corresponding DNA sequences of CPPs were confirmed by next generation sequencing (NGS) at each round of screen.

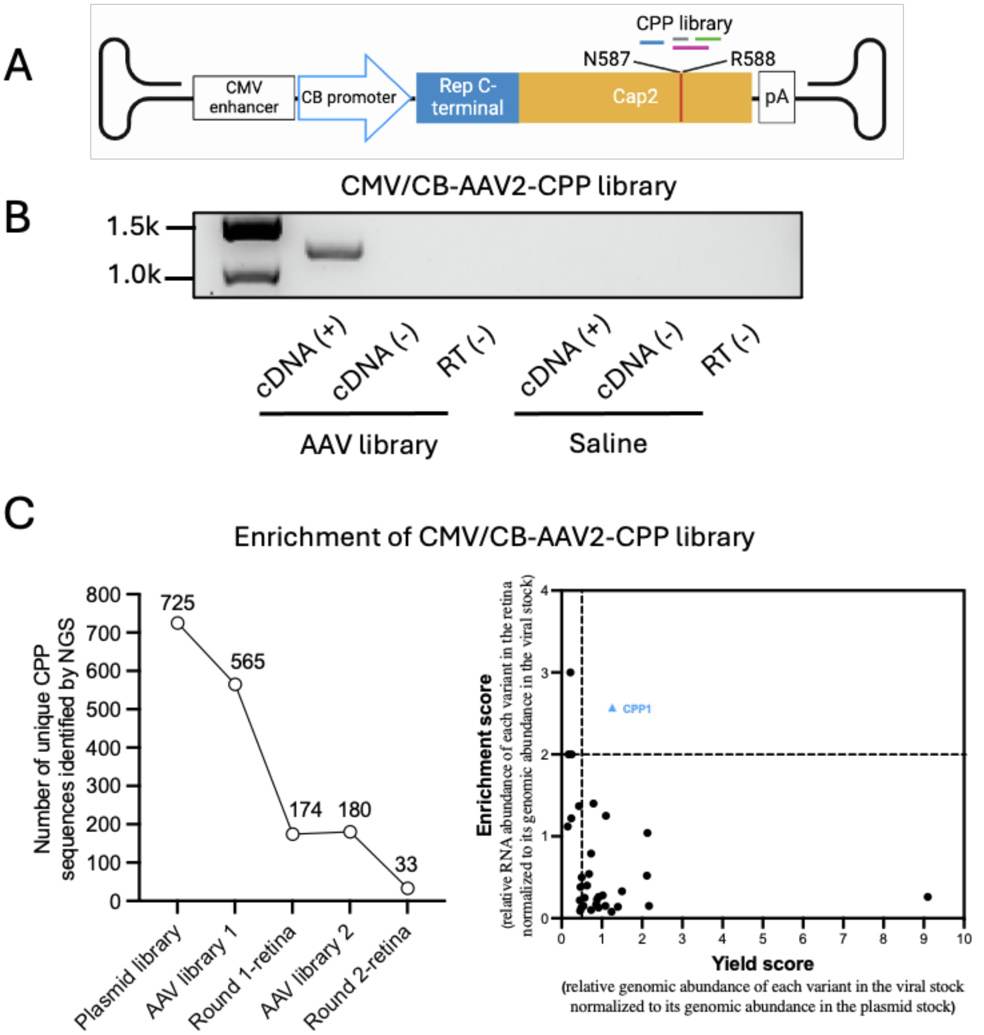

Figure S2. Identification of the leading AAV2 capsid variant enriched in the retina of mice using CMV/CB-AAV2-CPP library. (A) Schematic representation of the backbone plasmid construct containing the CPP library insert. (B) Representative RT-PCR results of recovered RNA of capsid variants from pooled retina/RPE tissues 28 days after injection of the CMV/CB-driven AAV library. (C) The reduction and enrichment of CPP variants during each round of selection. (D) Identification of AAV2.CPP1 as the leading capsid variant after the second round of selection based on enrichment and yield scores.

A

Viral vector name	Titer (vg/mL)	Packaging yield relative to AAV2 vector (%)
ssAAV2.CB6.GFP	4.30E+12	100%
ss7m8.CB6.GFP	1.54E+13	358%
ssAAV2.CPP1.CB6.GFP	2.54E+13	591%
ssAAV2.GRK1.H2BGFP	1.96E+12	100%
ss7m8.GRK1.H2BGFP	8.48E+12	433%
ssAAV2.CPP1.GRK1.H2BGFP	7.89E+12	403%
scAAV2.CB6.GFP	1.72E+12	100%
sc7m8.CB6.GFP	3.39E+12	197%
scAAV2.CPP1.CB6.GFP	1.29E+13	750%

B

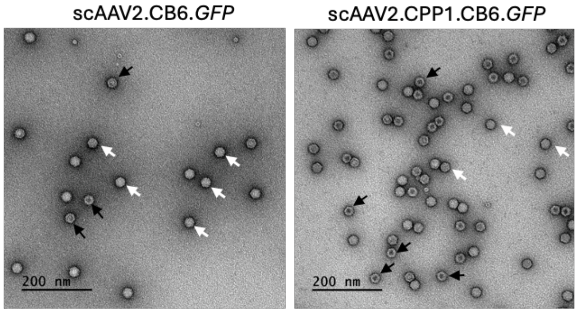

C

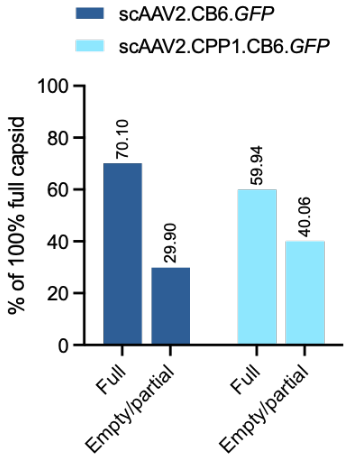

Figure S3. Engineered AAV2.CPP1 capsid can efficiently package AAV vectors with iodixanol gradient purification. (A) Titres of each AAV vectors packaged by AAV2, AAV2.7m8 and AAV2.CPP1 capsids. (B) Representative transmission electron microscopy images of scAAV2.CB6.*GFP* and scAAV2.CPP1.CB6.*GFP*. White and black arrows indicate full virions and completely or partially empty virions, respectively. (C) Quantification analysis of full/empty capsid ratio of each vector.

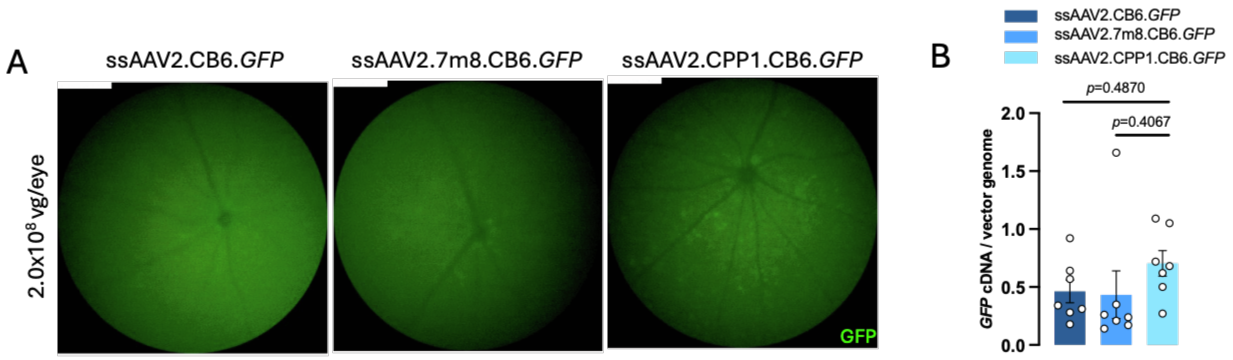

Figure S4. Retinal transduction profile of the single-stranded AAV2.CPP1 vector in adult mice at low dose. (A) Representative fluorescence fundus images of adult C57BL/6 mice four weeks after intravitreal injection with a low dose (2.0×10^{8} vg/eye) of single-stranded AAVs. (B) Quantification of genomic DNA levels of GFP in mouse retinas four weeks post-injection of low dose ssAAVs.

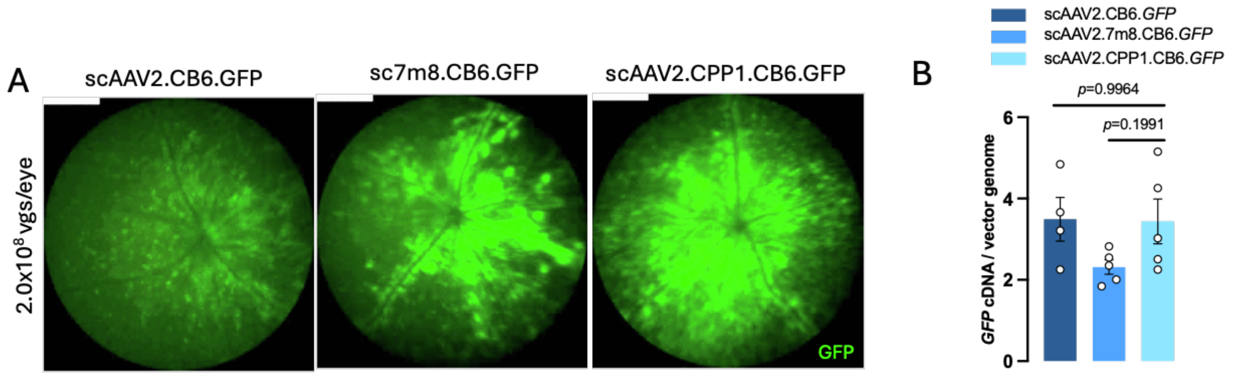

Figure S5. Retinal transduction profile of the self-complementary AAV2.CPP1 vector in adult mice at low dose. (A) Representative fluorescence fundus images of adult C57BL/6 mice four weeks after intravitreal injection with a low dose (2.0×10^{8} vg/eye) of single-stranded AAVs. (B) Quantification of genomic DNA levels of GFP in mouse retinas four weeks post-injection of low dose scAAVs.

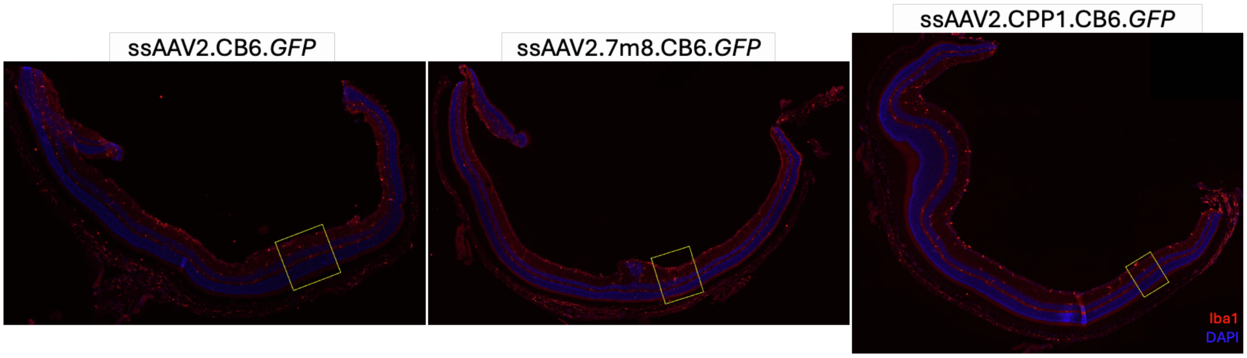

Figure S6. Full images of Figure 4A.
